# Quantum Dot-Based Screening Identifies F3 Peptide and Reveals Cell Surface Nucleolin as a Therapeutic Target for Rhabdomyosarcoma

**DOI:** 10.3390/cancers14205048

**Published:** 2022-10-14

**Authors:** Dzhangar Dzhumashev, Andrea Timpanaro, Safa Ali, Andrea J. De Micheli, Kamel Mamchaoui, Ilaria Cascone, Jochen Rössler, Michele Bernasconi

**Affiliations:** 1 Department of Pediatric Hematology and Oncology, Inselspital, Bern University Hospital, 3010 Bern, Switzerland; 2 Department for BioMedical Research (DBMR), University of Bern, 3008 Bern, Switzerland; 3 Graduate School for Cellular and Biomedical Sciences, University of Bern, 3012 Bern, Switzerland; 4 Department of Oncology, University Children’s Hospital Zurich, 8032 Zurich, Switzerland; 5 Children’s Research Center, University Children’s Hospital Zurich, University of Zurich, 3032 Zurich, Switzerland; 6 Centre de Recherche en Myologie, Institut de Myologie, INSERM, Sorbonne Université, F-75013 Paris, France; 7 IMRB, INSERM, University Paris Est Creteil, 94010 Creteil, France; 8 AP-HP, Groupe Hospitalo-Universitaire Chenevier Mondor, Centre d’Investigation Clinique Biothérapie, 94010 Créteil, France

**Keywords:** active targeting, quantum dots, nucleolin, F3 peptide, aptamer, rhabdomyosarcoma, Saporin toxin

## Abstract

**Simple Summary:**

Rhabdomyosarcoma accounts for more than 50% of all soft tissue sarcomas in childhood and adolescence. Despite intensified multimodality treatment, prognosis is extremely poor, with an overall survival rate of approximately 20% in the advanced stage. Therefore, there is an urgent need for targeted treatment options to improve overall survival rates, and to limit long-term side effects. Tumor-targeting peptides offer the possibility to deliver drugs selectively to the tumor site. Here, we select F3 as the best rhabdomyosarcoma-targeting peptide among a panel of 20 different tumor-targeting peptides. F3 peptide showed strong and specific binding to rhabdomyosarcoma, but not to normal cells, and efficient internalization with effective cytoplasmic delivery of the toxin Saporin. We show that nucleolin, the target of F3 peptide, is expressed on the surface of rhabdomyosarcoma cells. F3 peptide is a promising candidate for targeted drug delivery to rhabdomyosarcoma, e.g., by targeting drug-loaded nanoparticles.

**Abstract:**

Active drug delivery by tumor-targeting peptides is a promising approach to improve existing therapies for rhabdomyosarcoma (RMS), by increasing the therapeutic effect and decreasing the systemic toxicity, e.g., by drug-loaded peptide-targeted nanoparticles. Here, we tested 20 different tumor-targeting peptides for their ability to bind to two RMS cell lines, Rh30 and RD, using quantum dots Streptavidin and biotin-peptides conjugates as a model for nanoparticles. Four peptides revealed a very strong binding to RMS cells: NCAM-1-targeting NTP peptide, nucleolin-targeting F3 peptide, and two Furin-targeting peptides, TmR and shTmR. F3 peptide showed the strongest binding to all RMS cell lines tested, low binding to normal control myoblasts and fibroblasts, and efficient internalization into RMS cells demonstrated by the cytoplasmic delivery of the Saporin toxin. The expression of the nucleophosphoprotein nucleolin, the target of F3, on the surface of RMS cell lines was validated by competition with the natural ligand lactoferrin, by colocalization with the nucleolin-binding aptamer AS1411, and by the marked sensitivity of RMS cell lines to the growth inhibitory nucleolin-binding N6L pseudopeptide. Taken together, our results indicate that nucleolin-targeting by F3 peptide represents a potential therapeutic approach for RMS.

## 1. Introduction

Pediatric soft tissue sarcomas account for about 7% of all malignancies in children and young adults [1]. Rhabdomyosarcoma (RMS) is the most common soft tissue sarcoma of childhood [2]. Histology separates two major subtypes: embryonal or fusion-negative (FN-RMS, 60–70%), and alveolar or fusion-positive (FP-ARMS, 20–30%). FN-RMS is associated with a better prognosis than FP-RMS. Most aggressive FP-RMS tumors carry one of the two characteristic chromosomal translocations, the t(2;13)(q35;q14) or the t(1;13)(p36;q14), that result in the expression of a PAX3-FOXO1 and PAX7-FOXO1 fusion transcription factor, respectively [3]. Translation products of these gene fusions lead to increased expression of a number of oncogenes and are responsible for the aggressive phenotype of ARMS. Despite progress and intensified multimodality treatment, prognoses for these pediatric tumors are extremely bad, with an overall survival as low as 20% in the advanced stage [4,5]. Moreover, long-term toxicities of the intense chemotherapy/radiation therapy regimens are now becoming more evident with improving survival [6,7]. Therefore, new therapies are desperately needed for children and young adults with high-risk and recurrent solid tumors.

Nanocarriers loaded with drugs can decrease systemic side effects by passive accumulation in tumors through the enhanced permeation and retention (EPR) effect, a phenomenon first described by Maeda et al. [8,9]. However, the clinical translation of drug delivery by nanocarriers has not yet met the expectations [10,11]. Active targeting of nanocarriers to the tumor site by tumor-specific ligands promises to increase the therapeutic effect, while decreasing the systemic toxicity [12]. Selected peptides showing high affinity for diseased cells or tissue can indeed be conjugated to nanoparticles and bring their cargo to the tumor in a relatively selective fashion [13,14].

We previously selected RMS-targeting peptides by in vitro and in vivo phage display and tested them for their potential to increase vincristine-loaded liposomes’ accumulation in RMS mouse xenografts [15,16]. The peptide TmR was able to increase RMS binding of liposomes in vitro by 5.4-fold but did not increase tumor accumulation in vivo compared to non-targeted liposomes [15]. We hypothesized that a targeting peptide with a stronger binding to RMS might be needed to achieve increased accumulation in RMS tumors in vivo. Here, we selected from the literature peptides binding to receptors known to be expressed in RMS and compared their binding to RMS cell lines with peptides derived from our own screenings, using quantum dots (QD) as a model for nanoparticles with intrinsic fluorescence.

## 2. Materials and Methods

### 2.1. Materials

CdSe/ZnS quantum dots (QDs) Streptavidin nanocrystals with emission maxima at 525 nm (Q10143MP) or 605 nm (Q10101MP) and biotin (29129) were purchased from Thermo Fisher Scientific (Basel, Switzerland). Biotin-4-Fluorescein (AS-60654) was purchased from Eurogentec (Seraing, Belgium). Nonidet P-40 (ab142227) and Phalloidin-iFluor 488 (ab176753) were purchased from Abcam (Cambridge, UK). The 4′,6-diamidino-2-phenylindole (DAPI) and thiazolyl blue tetrazolium bromide (MTT) were purchased from Sigma Aldric (Buchs, Switzerland). All peptides were custom-ordered from GenScript (Leiden, Netherlands) or from GeneCust (Boynes, France) with the following modifications: Biotin-3x[Aminohexanoic acid-] at the N-terminus and amidation at the C-terminus, with HPLC purity over 95%, see Supplementary Table S1 for detailed information on peptides’ purity, scale, and sequences. Aptamer AS1411 [17] was synthesized by GenScript as a biotinylated oligonucleotide (Biotin-5’AminoC6-TTGGTGGTGGTGGTTGTGGTGGTGGTGG-3’), and all G were replaced with C in the negative control oligonucleotide (Neg.1411) [18]. Streptavidin-ZAP conjugates and free Saporin were purchased from Advanced Targeting Systems (San Diego, CA, USA). Human milk lactoferrin was from Sigma Aldrich. N6L pseudopeptide was kindly provided by Prof. Dr. José Courty (Université Paris-Est Créteil, Créteil, France). N6L hexavalently represents the pseudo-tripeptide Lysψ(CH2N)-Pro-Arg coupled to a polypeptide scaffold (Ac-**Lys**-Aib-Gly-**Lys**-Aib-Gly-**Lys**-Aib-Gly-**Lys**-Aib-Gly-**Lys**-Aib-Gly-**Lys**-Aib-Gly-CONH2), where Aib is 2-aminoisobutyric acid and ψ(CH2N) is a reduced peptide bond.

### 2.2. Cell Lines

Rh30, Rh4 (FP-RMS), RD, TTC-442 (FN-RMS), MRC-5 (human embryonal lung fibroblasts), and MDA-MB-231 (breast adenocarcinoma) were cultivated in Dulbecco’s modified Eagle’s medium (Bioconcept, Allschwil, Switzerland) with 10% fetal bovine serum (10270106, Thermo Fisher Scientific), 2 mM of L-glutamine, 100 U/mL of penicillin, and 100 µg/mL of streptomycin (Bioconcept). Mouse myoblasts C2C12 were cultivated with 20% FBS in DMEM. Cells were kindly provided by Prof. Beat Schäfer (University Children’s Hospital of Zurich). Immortalized human healthy primary myoblasts KM155C25Dist (referred to as myoblasts), kindly provided by the platform for immortalization of human cells MyoLine, from the Institut de Myologie (Sorbonne University, Paris, France), were cultured in Skeletal Muscle Cell Growth Medium (#C-23060, PromoCell, Heidelberg, Germany) with 5% fetal bovine serum, fetuin (bovine) 50 μg/mL, epidermal growth factor 10 ng/mL, basic fibroblast growth factor 1 ng/mL, insulin 10 μg/mL, and dexamethasone 0.4 μg/mL (PromoCell). Cells were fingerprinted by STR analysis (Microsynth, Balgach, Switzerland) and tested negative for mycoplasma.

### 2.3. Preparation and Quality Controls of Quantum Dots Streptavidin-Biotin Conjugates

CdSe/ZnS quantum dots Streptavidin (QD-SA) nanocrystals were incubated with biotinylated ligands (peptides or oligonucleotide) for at least 1.5 h at 4 °C with gentle agitation. The ratio of 50:1 ligand:QD was chosen as each QD nanocrystal has 5–10 Streptavidin molecules, according to the manufacturer’s description. Briefly, QD-SA were diluted to 100 nM in PBS (pH 7.4)/1% BSA and the biotinylated ligand was diluted to 5 μM in the same solution. After incubation, nonconjugated ligands were removed by size exclusion with PD SpinTrap G-25 with a cutoff < 5 kDa (GE28-9180-04, Sigma Aldrich). The concentration of the conjugates after size exclusion column was adjusted by measuring absorbance at 350 nm to calculate the concentration by the coefficient of extinction according to the Beer–Lambert law. Quality control of conjugation was performed by monitoring Biotin-4-Fluorescein quenching, as described in [19]. Briefly, upon binding of Biotin-4-Fluorescein to QD-Streptavidin nanocrystals, the quenching of the Biotin-4-Fluorescein signal can be measured at 450 nm. Successful conjugation of QD-SA with biotinylated molecules, or with free biotin, will prevent the quenching of the Biotin-4-Fluorescein signal (Supplementary Figure S1).

### 2.4. QD-Ligands Incubation with Cells

Quantification of QD-peptides conjugates binding to cells was performed by incubation with cells in suspension or with adherent cells. For incubation with cells in suspension, cells were detached with Accutase (Thermo Fisher Scientific) for 10 min at 37 °C, washed with PBS, and counted. Then, 100′000 cells were incubated in 100 μL of PBS/1% BSA with 20 nM QD-Ligand conjugates for 1 h at 37 °C, washed twice in PBS/1% BSA, and analyzed by FACS. For internalization experiments, 10′000 cells were plated in a 96-well plate. After 24 h, the medium was replaced with full growth medium containing 10 nM QD-peptides conjugates premixed by gentle agitation for 30 min. After 12 h of incubation at 37 °C and 5% CO_2_, cells were detached with Accutase, washed twice in PBS/1% BSA, and analyzed by FACS. QD-aptamer conjugates were incubated with adherent cells at a concentration of 10 nM and analyzed after 12 h of incubation at 37 °C and 5% CO_2_ by FACS.

### 2.5. Fluorescence-Activated Cell Sorting (FACS)

Measurements were performed with a CytoFLEX (Beckman Coulter), and the data were analyzed by doublets discrimination and determination of the geometric mean of the fluorescent intensity (MFI) using FlowJo v10.8 software (BD Life Sciences). The Fold Binding was calculated as the ratio of MFI values of QD-ligand conjugates over the nonconjugated QD-SA control. Statistical analysis was performed with GraphPad Prism software, version 8 (GraphPad Software, San Diego, CA). The statistical significance was assessed by the paired Student’s *t*-test and the Mann–Whitney test, with a threshold of *p* < 0.05. To detect cell surface receptors, cells were washed with PBS and detached with Accutase for 10 min at 37 °C. Then, 100′000 cells were incubated with polyclonal rabbit anti-NCL antibodies (20 µg/mL; N2662, Sigma) in 100 µL of PBS/1% BSA on ice for 1 h. After two washes with PBS/1% BSA, cells were stained with goat anti-rabbit IgG (H + L) Alexa488-conjugated (1:200; A11008, Thermo Fisher Scientific) for 30 min on ice. NCAM-1 receptor expression levels were determined with the semi-quantitative kit BD Quantibrite Beads (340495BD, Biosciences), following the manufacturer’s instructions. Cells were stained with mouse PE-conjugated anti-NCAM-1 antibodies (1:200; 318306, Biolegend) for 20 min on ice. Stained cells were processed with CytoFLEX as described above.

### 2.6. Fluorescence Microscopy

Cells were seeded on 8-well chamber cover-glass slides (80826, Ibidi, Grafelfing, Germany) at a density of 10′000 cells per well and incubated with 10 nM QD conjugates for 12 h at 37 °C and 5% CO_2_. After incubation, cells were washed with PBS, fixed with 2% PFA (Thermo Fisher Scientific) for 15 min at RT, and permeabilized with 0.1% Triton-X 100 for labeling with Phalloidin-iFluor 488. DAPI (D9542, Sigma Aldrich) in TBS was added for 2 min before mounting with a compatible medium (50001, Ibidi). Fluorescence images were acquired with a Leica 4000D microscope with Cy3 channel at 590 ± 23 nm to detect QD605. Acquired images were further processed by GIMP v2.10.0-RC1 (http://gimp.org).

### 2.7. ZAP-Conjugates’ Preparation and Cell Viability Assay

For internalization and the cargo delivery test, ZAP-conjugates were prepared for selected peptides. For conjugation, Streptavidin ZAP (Advanced Targeting Systems, Carlsbad, CA, USA) was incubated with biotinylated peptides at a ratio of 1:4. After 30 min of incubation at room temperature (25 °C) with gentle agitation, ZAP-conjugates were added to 2500 fast-growing cells (RD) or 5’000 slower growing (Rh30, Rh4, TTC-442) cells seeded in a 96-well plate. Three-fold dilution of initial stock was performed to obtain the series of decreasing concentrations of ZAP-conjugates (54, 18, 6, 2, 0.67, 0.22, 0.074, 0.0247 nM). ZAP-conjugates were incubated for 48 h. Viability was measured using the MTT assay.

### 2.8. Lactoferrin Competition

For the competition experiment, dilutions of 2 μM of human lactoferrin (SRP6519, Sigma Aldrich) in PBS/1% BSA were preincubated with 100′000 RMS cells in suspension at room temperature for 15 min, and then 20 nM QD-F3 was added and incubated for 1 h at 4 °C. Binding was quantified by FACS analysis, as described above.

### 2.9. Treatment with Pseudopeptide N6L

To test the effect of N6L pseudopeptide on RMS cells, cells were plated at a density of 5’000 cells per well in a 96-well plate and incubated with N6L for 72 h at 37 °C and 5% CO_2_. Concentrations of N6L from 0 to 100 μM were tested. The MDA-MB-231 cell line was used as a positive control sensitive to N6L [20]. The MTT assay was performed after 72 h.

### 2.10. MTT Cell Viability Assay

Cells in 96-well plates were incubated with 20 μL of MTT (5 mg/mL in PBS). After 4 h of incubation, the media and MTT reagent mixture were discarded and 150 μL of MTT solvent (4 mM HCl, 0.1% Nonidet P-40) in isopropanol was added. The absorbance at 490 nm was measured with a Tecan Sunrise microplate reader (Tecan Schweiz AG, Männedorf, Switzerland). Graphs were plotted with GraphPad Prism Software, version 8 (San Diego, CA, USA), using the nonlinear regression model. R^2^ was used to evaluate the goodness of fit to the model.

## 3. Results

### 3.1. Validation of QD-Peptides Streptavidin-Biotin Conjugates

Successful targeting of different cancer types has been reported with different tumor-targeting peptides (reviewed in [14]). Here, we set out to compare our own RMS-targeting peptides RMS-P3, TmR, RMS-I, and RMS-II with tumor-targeting peptides selected from the literature, to identify the best possible RMS-targeting peptide. We included peptides specific for surface receptors known to be expressed on RMS cells (NCAM-1, EGFR, CB1, TFR1, and uPAR; see Supplementary Table S2 for an overview of the literature), or reported to successfully target other tumors, tumor blood vessels, or tumor lymphatic vessels (Nucleolin, CD13, p32). We also included integrin α_v_β_3_-targeting peptides (CRGDS, cRGDyK) since integrin α_v_β_3_ is one of the most investigated targets [21,22] and we previously showed successful RMS-targeting with RGD-based peptides [23,24] (Table 1). Expression of the targeted receptor was additionally verified by analysis of publicly available expression data (Supplementary Figures S1–S3).

Peptides-conjugated quantum dots (QD) represent a powerful fluorescent probe allowing the estimation of ligand-mediated binding and internalization of nanoparticles [48]. We took advantage of commercially available QD Streptavidin (QD-SA) complexes, which are especially useful for stable conjugation with biotinylated ligands. First, to verify QD-SA surface coating with biotin conjugation, Biotin-4-Fluorescein was chosen as a fluorescent labeling molecule. Biotin-4-Fluorescein is quenched in proximity to Streptavidin (avidin) molecules [19], and therefore titration of Biotin-4-Fluorescein with QD-peptides conjugates could reveal the remaining vacant biotin sites (Supplementary Figure S4).

### 3.2. Screening of Peptide-Ligands Using QD Fluorescent Probe

**Figure 1:**
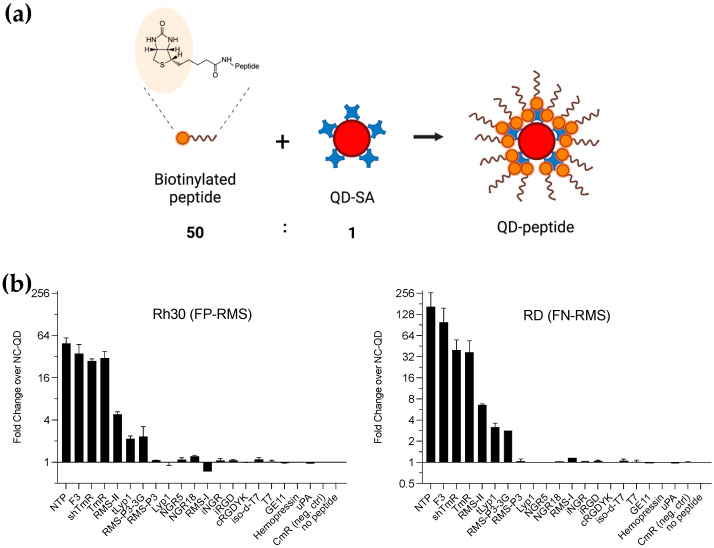
Scheme of QD-peptides conjugates’ preparation and binding to RMS cells. (**a**) Biotinylated peptides are incubated with QD-SA (605 nm) at a molar ratio of 50:1. After incubation, unbound peptides are removed by size exclusion chromatography with a 5 kDa cutoff. QD are expected to carry 5–10 Streptavidin (SA) molecules and can therefore incorporate up to 15–30 peptides. The estimated size of labeled QD-peptides conjugates is around 10 nm. (**b**) Binding of the selected QD-peptides conjugates to RMS cell lines RD (FN-RMS) and Rh30 (FP-RMS) was measured by FACS. 100′000 RMS cells were detached with Accutase and incubated in suspension with 20 nM QD-peptides conjugates, or nonconjugated QD-SA as a control, for 1 h at 37 °C with Rh30 cells (left panel) and RD cells (right panel). The fold-binding was calculated as the ratio between the geometric fluorescent mean intensity (FMI) of cells incubated with QD-peptides conjugates and the FMI of non-conjugated QD-SA from three independent experiments.

We included the biotinylated TAT peptide (YGRKKRRQRRR) as a positive control for successful conjugation of QD-SA and binding to RMS cells, and non-conjugated QD-SA and QD-CmR (non-binding peptide derived from TmR) as negative controls. Biotinylated peptides were conjugated to QD-SA by incubation peptides with QD-SA at a ratio of 50:1 (Figure 1). Unconjugated peptides were removed by size exclusion chromatography. The binding of QD-peptides conjugates to RMS cells was investigated on Rh30 cells (FP-RMS) and RD cells (FN-RMS) in suspension. We initially compared the binding of 20 nM QD-TAT conjugates incubated with adherent RMS cells for 2 h or with RMS cells in suspension. We did not observe a significant difference between incubation with adherent cells and cells in suspension. Therefore, all experiments for quantitative evaluation of QD-peptides’ binding by FACS were performed on RMS in suspension, as this allowed us to use less QD-peptides conjugates. QD-TAT always showed the highest fluorescence, between 100- and 450-fold over QD-SA.

QD-NTP and QD-F3 conjugates, targeting NCAM-1 and nucleolin, respectively, consistently showed the strongest binding to Rh30 cells (49.4- and 35.4-fold) and RD cells (165.0- and 98.5-fold). Our RMS-targeting peptides shTmR and TmR, modified versions of RMS-P3, also bound surprisingly strongly to Rh30 (27.8- and 30.4-fold, respectively) and to RD (40.0- and 36.9-fold, respectively). RMS-II, tLyp1, and RMS-P3-3G showed a weaker but significant binding to Rh30 and RD cells between 2.2- and 6.6-fold. All other peptides did not show any significant binding at the conditions chosen for these experiments, with a 20 nM concentration of QD-peptides conjugates.

**Figure 2:**
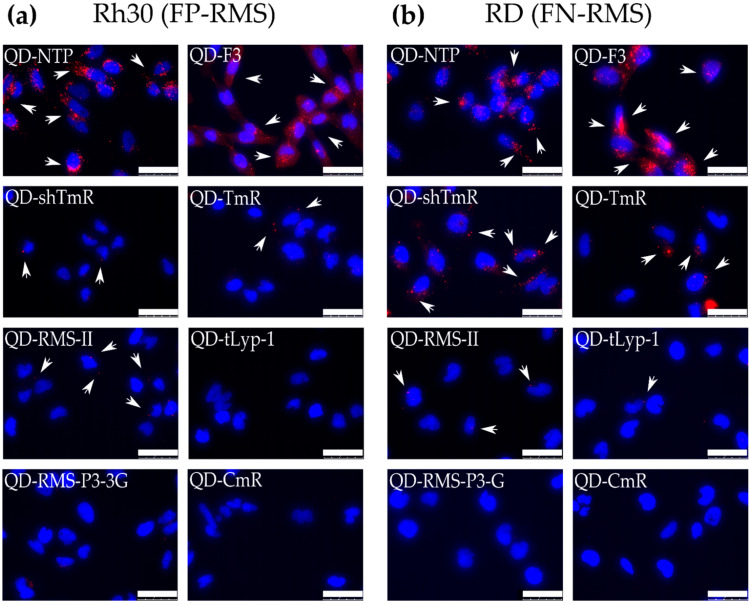
Internalization of QD-peptides conjugates into RMS cell lines. The QD-peptides conjugates that showed a strong biding to RMS cells by FACS were tested by fluorescent microscopy for internalization. (**a**) Rh30 cells and (**b**) RD cells were incubated for 12 h at 37 °C with 20 nM QD-peptides, fixed, and analyzed. CmR peptide was used as a negative control. Scale bars are 50 μm. Blue shows labeling of nuclei with DAPI, red shows QD605. White arrows point to internalized QD aggregates. Scale bar 50 μm.

To further assess binding and internalization in a qualitative manner, we performed fluorescent microscopy. All peptides with the binding fold change higher than 2 were tested in at least two RMS cell lines, the FP-RMS Rh30 and the FN-RMS RD. QD-peptides conjugates were incubated at 20 nM for 12 h with the cells, and after washing and fixation, cells were analyzed by fluorescence microscopy. NTP and F3 QD-peptides conjugates showed the strongest signals and displayed a clear internalization of QD into RMS cells (Figure 2). RMS-P3-derived peptides shTmR and TmR, and the peptide RMS-II, were also able to mediate QD internalization, however, to a much lower extent. Internalized QS were not or barely detectable for peptides tLyp1 and RMS-P3-3G. These data confirm the quantitative binding data obtained with FACS and indicate that NTP and F3 are the best candidates for targeting of RMS cells.

**Figure 3:**
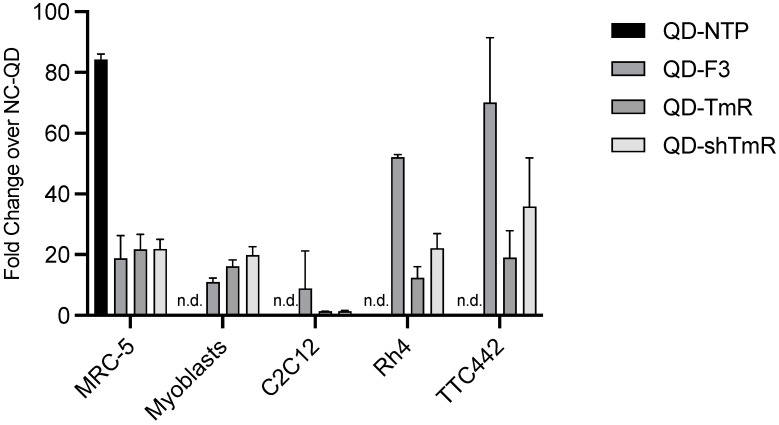
Binding of QD-NTP, QD-F3, QD-TmR, and QD-shTmR to RMS cells and normal fibroblasts and myoblasts. 100′000 cells were detached with Accutase and incubated in suspension with 20 nM QD-peptides conjugates, or nonconjugated QD-SA as a control, for 1 h at 37 °C, with lung embryonal fibroblasts MRC-5, human myoblasts, and mouse myoblasts C2C12, FP-RMS Rh4, and FN-RMS TTC-442. The fold-binding was calculated as the ratio between the geometric fluorescent mean intensity (FMI) of cells incubated with QD-peptides conjugates and the FMI of non-conjugated QD-SA from three independent experiments. QD-NTP bound efficiently to MRC-5, which are negative for NCAM-1, the target of NCR-peptide; therefore, QD-NTP were not tested on the other cells. n.d.: Not done.

Next, we evaluated the binding of the best peptides to control normal cells: human embryonal fibroblasts (MRC5), primary human myoblasts (KM155C25Dist), and mouse myoblast C2C12, as well as to additional RMS cell lines (FP-RMS Rh4 and FN-RMS TTC-442). The peptide NTP revealed a very strong binding to MRC-5 (Figure 3), which is negative for surface expression of its target NCAM-1 (Supplementary Figure S5). This result questions the specificity of the NTP peptide. Therefore, the NTP peptide was omitted from the following experiments. The F3 peptide showed moderate binding to MRC-5 and primary myoblasts, but strong binding to the RMS cell lines Rh4 and TTC-442. TmR and shTmR peptides showed a moderate biding to control cells, and to RMS cells. In conclusion, these results confirm the strong targeting potential of F3 peptides and indicate that TmR and shTmR are specific for RMS but are less strong than F3.

### 3.3. Peptide-Mediated Internalization and Cytoplasmic Payload Delivery

**Figure 4:**
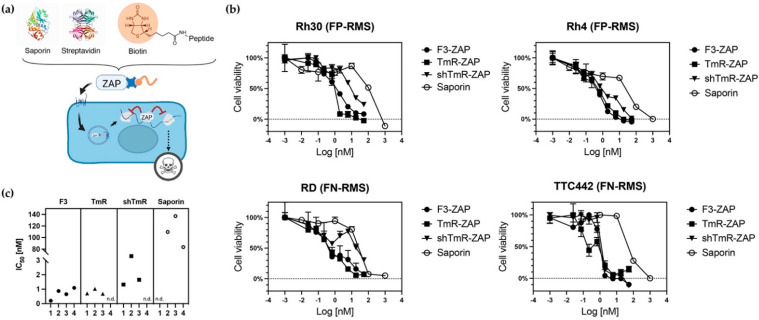
Efficient cytoplasmic delivery of Saporin by selected RMS-targeting peptides. (**a**) Structure and mechanism of action of peptides-Saporin conjugates. Ribosomes’ inhibition results in cell death. (**b**) RMS cells were treated with increasing concentrations of peptides-Saporin conjugates for 48 h, and cell viability was measured by the MTT assay. (**c**) Summary of IC_50_ values calculated for Saporin alone and the different peptides-Saporin conjugates. 1: Rh30, 2: Rh4, 3: RD, 4: TTC-442. n.d.: Not determined, i.e., the fit of the nonlinear regression model’s curve R^2^ was <0.9 and IC_50_ could not be determined. Experiments were performed once in triplicates.

To assess the ability of the selected peptides to bind, internalize, and deliver a payload in the cytoplasm in RMS cells, we conjugated biotinylated peptides to the commercially available Streptavidin-ZAP, consisting of Saporin chemically conjugated to Streptavidin (Figure 4a). Saporin is a ribosome-inactivating protein first isolated from *Sapinaria officinalis* [49], which is useful to evaluate immunotoxin strategies and to delete specific cells in vivo [50]. Saporin depurinates a specific nucleotide in the ribosomal RNA 28S (A_4324_), thus irreversibly blocking protein synthesis. Without a targeting moiety, Saporin cannot efficiently internalize and has a relatively high IC_50_ (Figure 4c). A decrease in the IC_50_ of targeted Saporin indicates successful intracellular uptake and enhanced lysosome escape into the cytoplasm. The most promising peptides, F3, TmR, and shTmR, were tested. NTP was not included due to its potential lack of specificity observed on control cells (Figure 3). FP-RMS cells Rh30 and Rh4, as well as FN-RMS cells RD and TTC-442, were incubated with increasing concentrations of peptides-ZAP conjugates. Most of them (F3-ZAP, shTmR-ZAP, TmR-ZAP) led to an IC_50_ decrease of around 100-fold compared to free Saporin in the RMS cells tested (Figure 4b,c). The negative control peptide CmR displayed a weak toxicity, insufficient to calculate IC_50_ in the same range of concentrations as the other peptide-ZAP conjugates. None of the tested free peptides were toxic even at the highest tested concentrations.

F3 peptide and TmR peptide consistently showed the most efficient internalization of Saporin on the RMS cell lines tested. Since F3 peptide showed the most efficient and specific binding to RMS cells, as measured by FACS, we selected F3 peptide as our lead candidate and proceeded with the validation of the expression of its target, nucleolin, on RMS cells.

### 3.4. Validation of Nucleolin as a Specific Target for RMS

Nucleolin was identified as a ligand for F3 peptide by affinity purification from MDA-MB-435 lysates, and the localization of nucleolin on the cell surface was demonstrated with a polyclonal rabbit antibody (NCL3) raised against amino acids 221–232 of human nucleolin, and with a mouse monoclonal antibody (MS-3) [25]. The polyclonal antibody which gave the strongest signal is not available anymore, but we tested the MC-3 mAb on RMS cells. From FACS, we did not observe any significant binding of the anti-nucleolin MC-3 antibody to surface nucleolin on RMS cells. We could observe a shift in the FACS signal when using a polyclonal rabbit antibody only in RD cells (Supplementary Figure S6). Considering the weak shifts in FACS signals observed by us and by others [25,51,52,53,54], we decided to take another approach to validate the presence of nucleolin on the surface of RMS cells.

**Figure 5:**
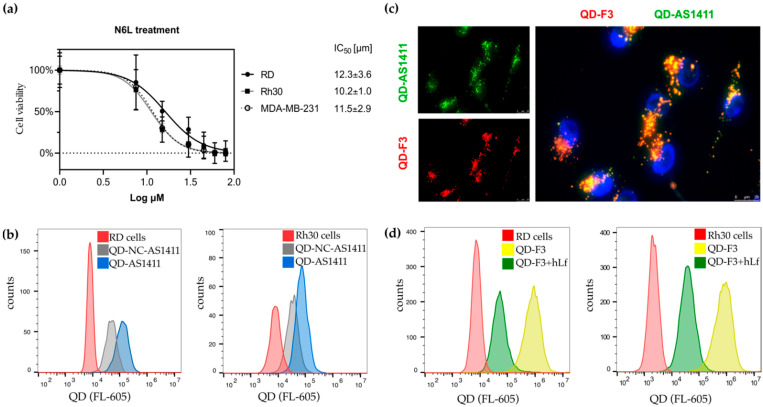
Validation of nucleolin presence on the surface of RMS cells by alternative nucleolin ligands. (**a**) The nucleolin antagonist N6L reduces RMS cells’ viability in culture. RMS cells (RD, Rh30) as well as MDA-MB-231 were incubated with increasing concentrations of N6L for 72 h. Cell viability was determined by the MTT assay, and the IC_50_ of N6L was calculated. (**b**) The biotinylated aptamer AS1411, or its negative control NC-AS1411, were conjugated to QD-SA, and incubated overnight at 20 nM with RMS cells. QD-AS1411 shows increased binding to RMS cells compared to the mutated oligonucleotide QD-NC-AS1411. (**c**) 10 nM QD(525)-AS1411 and QD(605)-F3 were co-incubated with RD cells overnight. After washing and fixation, cells were analyzed with fluorescence microscopy. The image shows colocalization of QD-F3 (red) and QD-AS1411 (green) after binding and internalization on RD cells. (**d**) Co-incubation with human lactoferrin, a high-affinity natural ligand of nucleolin, leads to decreased binding of QD-F3 to the RMS cells RD and Rh30. Cells were detached, pre-incubated with 2 μM lactoferrin for 20 min, and then co-incubated with 20 nM QD-F3 on ice for 1 h.

We took advantage of two well-characterized anti-nucleolin synthetic ligands, namely the pseudopeptide NucAnt 6L (N6L [55]) and the aptamer AS1411 [56]. Both promote cell death in tumor cells and N6L has reached clinical trials in solid tumors (NCT01711398) and AS1411 in renal cell carcinoma (NCT00740441). Pseudopeptide N6L was developed as a modification of the pseudopeptide HB-19 [57,58], where a LysΨ(CH_2_N)-Pro-Arg pseudo-tripeptide was conjugated to the ε-NH_2_ of Lys, a part of the helical matrix composed of six repeats of Lys-Aib-Gly. The reduced peptides bond (Ψ(CH_2_N)) in the pseudo-tripeptide promotes stability against endogenous proteases in serum. N6L is cytotoxic at low micromolar concentrations (IC_50_) for a wide range of tumors [20]. Therefore, we tested the effect of N6L on RMS cells. Increasing concentrations of N6L were added to RMS cells RD and Rh30, as well as to the breast adenocarcinoma cell line MDA-MB-231, for which a growth inhibition 50% (GI_50_) of 20 µM is reported [20]. After 72 h, the number of viable cells was quantified by the MTT assay. These experiments revealed a strong toxicity of N6L on RMS cells, comparable to MDA-MB-231. N6L displayed an IC_50_ of 12.3 and 10.2 μM for RD and Rh30 cells, respectively. For MDA-MB-231, used as a positive control, the IC_50_ was 11.5 µM (Figure 5a). Therefore, RMS cell lines have an IC_50_ in a low micromolar range as the N6L-susceptible cell line MDA-MB-231, suggesting nucleolin expression also on the surface of RMS cells.

G-quadruplex oligonucleotide AS1411, previously known as AGRO100, has been discovered as a first aptamer with an antiproliferative effect on cancer cells (reviewed in [56]). The mechanism of the cytotoxic effect of the aptamer AS1411 was identified in the blocking of nuclear factor-κB essential modulator (NEMO) at the protein level [59]. In addition to NF-κB signaling abrogation, blocking of Bcl-2 mRNA at the protein-mRNA level was discovered independently in human breast cancer cell lines [60]. The mechanism of cellular internalization proposed is a multi-step process with macropinocytosis of AS1411 and consequent activation of nucleolin on the cell surface [61]. AS1411 has been used for targeted drug delivery with PEGylated liposomes [62], or on DNA nanorobots [63]. QD conjugated with AS1411 have been successfully used for imaging and ligand-binding analysis in vitro [18,64,65,66]. Here, we used QD-SA conjugates and labeled them with biotinylated AS1411. As a negative control, we used an oligonucleotide sequence with all G mutated to C [18]. After overnight incubation, cell binding was quantified by FACS (Figure 5b). QD-AS1411 bound to RD cells 8.8-fold more, and to Rh30 cells 2.2-fold more than the control QD-NC-AS1411. In addition, we generated QD-AS1411 and QD-F3 with different emission maxima (QD525, green and QD605, red, respectively) and co-incubated them with RD cells at 10 nM each overnight. A clear colocalization of QD-F3 and QD-AS1411 was visible by fluorescence microscopy (Figure 5c), but not between QD-F3 and QD-NC-AS1411.

Several natural ligands of cell surface nucleolin have been identified, such as midkine [67] and human lactoferrin [64]. Nucleolin-derived peptides were tested for their interaction with lactoferrin by SPR [64]. The nature of the interaction suggests that nucleolin is a high-affinity receptor for naturally occurring lactoferrin. Therefore, we used lactoferrin to compete with the binding of QD-F3 to nucleolin and to test if some level of QD-F3 binding decrease can be achieved by competition and consumption of surface nucleolin by excess of lactoferrin in the media. After a short pretreatment of RMS cells RD or Rh30 with 2 μM of lactoferrin for 20 min, 20 nM QD-F3 and 2 μM of lactoferrin were co-incubated for 1 h on ice with RMS cells. After washing, QD-F3 binding was quantified by FACS. A clear decrease of QD-F3 binding could be observed for both RMS cell lines RD and Rh30 upon co-incubation with excess lactoferrin, suggesting a competition for receptor-specific binding and internalization (Figure 5d).

Taken together, these results show that synthetic ligands of nucleolin (pseudopeptide N6L and aptamer AS1411), as well as the natural ligand of nucleolin lactoferrin, can compete with QD-F3 for binding to RMS cells, supporting the notion that F3-binding to RMS cells is mainly mediated by the interaction with nucleolin.

## 4. Discussion

In this study, we screened 20 peptides targeting 12 different receptors for their binding to two RMS cell lines. We identified the nucleolin-targeting peptide F3 as the most effective ligand to mediate binding and internalization of QD nanoparticles and for delivery of the toxin Saporin to RMS cells. We validated surface expression of nucleolin on RMS cells by competition of F3-binding to RMS cells with the nucleolin ligands aptamer AS1411 and lactoferrin, colocalization of QD-F3 and QD-AS1411 in RMS cells, and by the high sensitivity of RMS cells to the cytotoxic nucleolin-binding pseudopeptide N6L.

The Furin-targeting peptide TmR, described previously by our group, and its shorter version shTmR missing a GGG spacer, were also very specific and efficient in delivering QD and Saporin to RMS cells, but bound about 2- to 5-fold less strongly to RMS cells than F3 peptide. The RMS-II peptide, with sequence homology to Lyp-1, previously selected by phage display on RMS cell lines, also performed well, but bound 5- to 10-fold less than F3 peptide. The NCAM-1-targeting peptide NTP bound to RMS cells extremely well, probably also owing to the high expression of NCAM-1 in RMS cells, but unfortunately also significantly bound to the NCAM-1-negative cells MRC-5, suggesting that the NTP peptide might not be completely specific for NCAM-1. For this reason, the NTP peptide was not further pursued.

QD-Streptavidin conjugates together with biotinylated ligands represent a versatile platform for quantitative and qualitative screening of tumor-targeting peptides in a robust and reproducible way. The biotin–Streptavidin interaction is very stable and has a dissociation constant (Kd) of 10–14. Incubation of an excess of biotinylated peptides with QD-Streptavidin, followed by size exclusion purification, allowed to have QD-peptide conjugates with all biotin-binding sites saturated, as verified by Biotin-4-Fluorescein quenching. The only limitation is the price of QD conjugates, that allows to use them only at concentrations around 20 nM with reasonable costs. A 20 nM concentration is quite low, but it favors the stringent selection of peptides with a very high affinity, or with a very high number of target receptors on the surface, both ideal qualities for targeting peptides. This might in part explain why so many of the tested peptides—several of which were already shown to be able to deliver nanoparticles to tumors in mouse models—performed so poorly in our screening. A negative result does not exclude that these peptides can achieve efficient targeting at higher concentrations. The CendR peptides iRGD, iNGR, and tLyp-1 targeting Neuropilin-1, as well as peptides binding preferentially to angiogenic blood vessels (NGR, Lyp-1), are expected to be very efficient in vivo, and a lack of binding in vitro does not imply they will not be able to target RMS tumors in vivo.

In the present study, we used QD-peptides conjugates in 2D cell culture to evaluate binding to the cell-specific receptor. With the growing availability of 3D culture and organoid models as potential replacements for animal tumor models, QD-conjugates could be used for imaging and detection of cell populations overexpressing specific molecules on the surface, as recently shown for two different populations in spheroids formed from two cell lines [68]. Presumably, a similar approach could be utilized in clinically relevant models of tumor spheroids or patient-derived organoids. Exosomes play an important role for tumor development and could be used as biomarkers detected by QD-conjugates together with anti-exosome magnetic beads (e.g., anti-CD81), as shown for HER2-positive patient-derived exosomes [69]. Another source of biomarkers relevant for metastasis characterization are circulating tumor cells. Several reports exploited the fluorescent advantages of QD-conjugates for detection of low numbers of circulating tumor cells [70,71,72].

Nucleolin was identified as a ligand for F3 peptide by affinity purification from MDA-MB-435 lysates [31]. Transient silencing of nucleolin expression by siRNA decreased binding saturation of F3 on MDA-231-MB cells [73], and confirmed the specificity of other ligands for nucleolin [74,75]. We tried to knockout nucleolin by CRISPR/Cas9 in RD and Rh4 cells, but we did not succeed in selecting viable clones, suggesting that complete nucleolin knock-down is lethal (Dzhangar Dzhumashev, unpublished results).

Nucleolin is abundantly expressed in exponentially growing cells, such as cancer cells, and is mainly located in the nucleus, where it controls various important cellular functions. Nucleolin is transported to the cell surface as a part of the shuttling mechanism [76,77]. Surface expression of nucleolin has been reported in many different cancer types, and its involvement in cancer has been extensively studied (reviewed in [78,79,80,81,82]). At the cell surface, nucleolin can act as a receptor for various viruses [83]. The detailed mechanism of internalization and subcellular fate of F3-functionalized nanoparticles has been intensively studied using polyacrylamide nanocarriers (NC) and comparing targeted NC, non-targeted NC, and F3 peptide alone. Clathrin-dependent endocytosis was shown to be the prevalent mechanism of internalization [84].

The presence of nucleolin on the surface of the FN-RMS cell line RD, also used in our study, is supported by the nucleolin-dependent infection by the Enterovirus 71 of RD [85]. Further evidence comes from the pulldown of surface nucleolin from the RD cell line with the HB-19 pseudopeptide, the precursor of N6L, and detection by immunoblotting [86]. Moreover, the anti-nucleolin aptamers AS1411 and iSN04 showed a potent growth inhibitory effect on RD cells [87]. The role of nucleolin in RMS remained unexplored until it was recently shown that nucleolin acts as a cofactor for the oncogenic transcription factor TBX3, also in the RD cell line [88]. Correlation between high nucleolin and TBX3 co-expression and poor survival was observed in sarcomas [88]. Importantly, the signaling axis c-Myc/AKT1/TBX3 has been identified as an important interventional target for embryonal RMS [89]. Finally, nucleolin was among the 80 most enriched proteins detected by proteomics analysis in extracellular vesicles from five RMS cell lines [90].

N6L is cytotoxic at low micromolar concentrations (IC_50_) for a wide range of cancer cell lines, such as renal carcinoma, glioblastoma, melanoma, acute lymphoblastic leukemia, and mammary gland adenocarcinoma. In vivo, N6L leads not only to the inhibition of tumor progression, but also affects vascular development [20,91,92]. Based on our results, we propose a high and dynamic presence of nucleolin on the cell surface that makes it an attractive target for RMS treatment with proapoptotic ligands such as AS1411 and N6L, or with antibody-drug conjugates (ADC) based on high-affinity monoclonal antibodies, scFv, or single-domain antibodies against nucleolin.

We previously tested the peptide TmR for its potential to increase vincristine-loaded liposomes’ accumulation in RMS mouse xenografts [15,16]. TmR was able to increase the binding of liposomes to RMS cells in vitro by 5-fold but did not further increase tumor accumulation in vivo compared to non-targeted liposomes, which accumulated 5-fold more (460-fold AUC_0-24h_) compared to free vincristine [15]. Here, we aimed at selecting a peptide with stronger binding to RMS cells than TmR, and F3 peptide bound to the four RMS cell lines tested 3- to 5-fold more than TmR peptide. Importantly, F3 peptide has been successfully used to target doxorubicin-loaded pH-sensitive liposomes to breast and mesothelioma tumors [93] and neuroblastoma [94] in mice. Taken together, these results support the use of F3 peptide to target vincristine-loaded liposomes to RMS.

## 5. Conclusions

In summary, we have identified F3 as an effective binding peptide to RMS cells, superior to the previously tested RMS-targeting peptides, and with high specificity that can be further developed into nanoparticles for targeted drug delivery to RMS.

## Figures and Tables

**Table 1:** Rhabdomyosarcoma cell surface proteins as potential peptide-ligands targets.

Targets Expressed on Tumor Blood and/or Lymphatic Vessels as Well as on Tumors			
Target	Peptide	Sequence	Ref.
Nucleolin	F3 (29 aa)	EPQRRSARLSAKPAPPKPEPKPKKAPAKK	[25,26,27]
CD13	NGR-5	CNGRC *	[28]
	NGR-18	CNGRCGVRSSSRTPSDKY	[29]
p32	Lyp-1	CGNKRTRGC	[30]
CD13, Neuropilin-1	iNGR	CRNGRGPDC	[31]
p32, Neuropilin-1,	tLyp-1	CGNKRTR	[32]
Integrin α_v_β_3,_ Neuropilin-1	iRGD	CRGDKGPDC	[33]
**Targets Expressed on Rhabdomyosarcoma**			
**Target**	**Peptide**	**Sequence**	**Ref.**
Integrin α_v_β_3_	Linear RGD	CRGDS	[34]
	Cyclic RGD	cRGDyK	[35,36,37]
	RMS-I	CQQSNRGDRKRC *	[38]
Furin	TmR	KRDRGGGCMGTINTRTRRC *	[15]
	shTmR	KRDRCMGTINTRTRRC *	This work
	RMS-P3-3G	GGGCMGTINTRTRRC *	[16]
	CmR (neg. ctr.)	KRDRGGGCMGTINTATAAC *	[15]
Neural Cell Adhesion Molecule 1 (NCAM-1)	NTP	ASKKPKRNIKA	[39,40,41]
Cannabinoid Receptor 1 (CB1)	Hemopressin	PVNFKFLSH	[42]
Epidermal Growth Factor Receptor (EGFR)/ErbB1	GE11	YHWYGYTPQNVI	[43]
Transferrin Receptor 1 (TFR1)	T7	HAIYPRH	[44,45]
	iso-d-T7	d-(HRPYIAH)	[46]
Urokinase Plasminogen Activator Receptor (uPAR)	uPA	VSNKYFSNIHWGC	[47]
Target undetermined	RMS-II	CMGNKRSAKRPC *	[38]

* Cyclization via disulfide bridge between underlined cysteines.

## Data Availability

All the data generated in this paper are available upon reasonable request to M.B.

## Supplementary Materials

The following are available online at https://www.mdpi.com/article/10.3390/cancers14205048/s1, Table S1: Peptides used in this study; Table S2: Published work supporting expression of selected targets in RMS cell lines or tumors; Figure S1: Targeted receptors’ expression in RMS cell lines, tumors, and skeletal muscles; Figure S2: Targeted receptors’ expression in RMS cell lines, tumors, and normal tissues; Figure S3: Expression of selected surface targets including nucleolin and p32 in RMS cell lines, tumors, and skeletal muscle; Figure S4: Quality control for conjugation of Streptavidin-QD and biotin-peptides by B4F-quenching; Figure S5: Surface expression of NCAM1 on RMS cell lines and control cells; Figure S6: Surface detection of nucleolin on RMS cells [95,96,97,98,99,100,101,102,103,104,105,106].

## References

[1] Ognjanovic S., Linabery A.M., Charbonneau B., Ross J.A. (2009). Trends in childhood rhabdomyosarcoma incidence and survival in the United States, 1975–2005. Cancer.

[2] Loeb D.M., Thornton K., Shokek O. (2008). Pediatric soft tissue sarcomas. Surg. Clin. N. Am..

[3] Barr F.G., Smith L.M., Lynch J.C., Strzelecki D., Parham D.M., Qualman S.J., Breitfeld P.P. (2006). Examination of gene fusion status in archival samples of alveolar rhabdomyosarcoma entered on the Intergroup Rhabdomyosarcoma Study-III trial: A report from the Children’s Oncology Group. J. Mol. Diagn..

[4] Malempati S., Weigel B.J., Chi Y.Y., Tian J., Anderson J.R., Parham D.M., Teot L.A., Rodeberg D.A., Yock T.I., Shulkin B.L. (2019). The addition of cixutumumab or temozolomide to intensive multiagent chemotherapy is feasible but does not improve outcome for patients with metastatic rhabdomyosarcoma: A report from the Children’s Oncology Group. Cancer.

[5] Shern J.F., Selfe J., Izquierdo E., Patidar R., Chou H.C., Song Y.K., Yohe M.E., Sindiri S., Wei J., Wen X. (2021). Genomic Classification and Clinical Outcome in Rhabdomyosarcoma: A Report From an International Consortium. J. Clin. Oncol..

[6] Punyko J.A., Mertens A.C., Gurney J.G., Yasui Y., Donaldson S.S., Rodeberg D.A., Raney R.B., Stovall M., Sklar C.A., Robison L.L. (2005). Long-term medical effects of childhood and adolescent rhabdomyosarcoma: A report from the childhood cancer survivor study. Pediatr. Blood Cancer.

[7] Owosho A.A., Brady P., Wolden S.L., Wexler L.H., Antonescu C.R., Huryn J.M., Estilo C.L. (2016). Long-term effect of chemotherapy-intensity-modulated radiation therapy (chemo-IMRT) on dentofacial development in head and neck rhabdomyosarcoma patients. Pediatr. Hematol. Oncol..

[8] Matsumura Y., Maeda H. (1986). A new concept for macromolecular therapeutics in cancer chemotherapy: Mechanism of tumoritropic accumulation of proteins and the antitumor agent smancs. Cancer Res..

[9] Hashida M. (2022). Advocation and advancements of EPR effect theory in drug delivery science: A commentary. J. Control Release.

[10] Park K. (2013). Questions on the role of the EPR effect in tumor targeting. J. Control Release.

[11] Sun D., Zhou S., Gao W. (2020). What Went Wrong with Anticancer Nanomedicine Design and How to Make It Right. ACS Nano.

[12] Nag O.K., Delehanty J.B. (2019). Active Cellular and Subcellular Targeting of Nanoparticles for Drug Delivery. Pharmaceutics.

[13] Araste F., Abnous K., Hashemi M., Taghdisi S.M., Ramezani M., Alibolandi M. (2018). Peptide-based targeted therapeutics: Focus on cancer treatment. J. Control Release.

[14] Roveri M., Bernasconi M., Leroux J.C., Luciani P. (2017). Peptides for tumor-specific drug targeting: State of the art and beyond. J. Mater. Chem. B.

[15] Roveri M., Pfohl A., Jaaks P., Alijaj N., Leroux J.C., Luciani P., Bernasconi M. (2017). Prolonged circulation and increased tumor accumulation of liposomal vincristine in a mouse model of rhabdomyosarcoma. Nanomedicine.

[16] Hajdin K., D’Alessandro V., Niggli F.K., Schafer B.W., Bernasconi M. (2010). Furin targeted drug delivery for treatment of rhabdomyosarcoma in a mouse model. PLoS ONE.

[17] Bates P.J., Kahlon J.B., Thomas S.D., Trent J.O., Miller D.M. (1999). Antiproliferative activity of G-rich oligonucleotides correlates with protein binding. J. Biol. Chem..

[18] Ko M.H., Kim S., Kang W.J., Lee J.H., Kang H., Moon S.H., Hwang D.W., Ko H.Y., Lee D.S. (2009). In vitro derby imaging of cancer biomarkers using quantum dots. Small.

[19] Mittal R., Bruchez M.P. (2011). Biotin-4-fluorescein based fluorescence quenching assay for determination of biotin binding capacity of streptavidin conjugated quantum dots. Bioconjug Chem..

[20] Destouches D., Page N., Hamma-Kourbali Y., Machi V., Chaloin O., Frechault S., Birmpas C., Katsoris P., Beyrath J., Albanese P. (2011). A simple approach to cancer therapy afforded by multivalent pseudopeptides that target cell-surface nucleoproteins. Cancer Res..

[21] Sheikh A., Alhakamy N.A., Md S., Kesharwani P. (2021). Recent Progress of RGD Modified Liposomes as Multistage Rocket Against Cancer. Front. Pharmacol..

[22] Ludwig B.S., Kessler H., Kossatz S., Reuning U. (2021). RGD-Binding Integrins Revisited: How Recently Discovered Functions and Novel Synthetic Ligands (Re-)Shape an Ever-Evolving Field. Cancers.

[23] Scherzinger-Laude K., Schonherr C., Lewrick F., Suss R., Francese G., Rossler J. (2013). Treatment of neuroblastoma and rhabdomyosarcoma using RGD-modified liposomal formulations of patupilone (EPO906). Int. J. Nanomed..

[24] Rengaswamy V., Zimmer D., Suss R., Rossler J. (2016). RGD liposome-protamine-siRNA (LPR) nanoparticles targeting PAX3-FOXO1 for alveolar rhabdomyosarcoma therapy. J. Control Release.

[25] Zhou M., Ghosh I. (2007). Quantum dots and peptides: A bright future together. Biopolymers.

[26] Christian S., Pilch J., Akerman M.E., Porkka K., Laakkonen P.M., Ruoslahti E. (2003). Nucleolin expressed at the cell surface is a marker of endothelial cells in angiogenic blood vessels. J. Cell Biol..

[27] Porkka K., Laakkonen P., Hoffman J.A., Bernasconi M., Ruoslahti E. (2002). A fragment of the HMGN2 protein homes to the nuclei of tumor cells and tumor endothelial cells in vivo. Proc. Natl. Acad. Sci. USA.

[28] Fonseca N.A., Gomes-da-Silva L.C., Moura V., Simoes S., Moreira J.N. (2014). Simultaneous active intracellular delivery of doxorubicin and C6-ceramide shifts the additive/antagonistic drug interaction of non-encapsulated combination. J. Control Release.

[29] Ellerby H.M., Arap W., Ellerby L.M., Kain R., Andrusiak R., Rio G.D., Krajewski S., Lombardo C.R., Rao R., Ruoslahti E. (1999). Anti-cancer activity of targeted pro-apoptotic peptides. Nat. Med..

[30] Curnis F., Cattaneo A., Longhi R., Sacchi A., Gasparri A.M., Pastorino F., Di Matteo P., Traversari C., Bachi A., Ponzoni M. (2010). Critical role of flanking residues in NGR-to-isoDGR transition and CD13/integrin receptor switching. J. Biol. Chem..

[31] Laakkonen P., Porkka K., Hoffman J.A., Ruoslahti E. (2002). A tumor-homing peptide with a targeting specificity related to lymphatic vessels. Nat. Med..

[32] Alberici L., Roth L., Sugahara K.N., Agemy L., Kotamraju V.R., Teesalu T., Bordignon C., Traversari C., Rizzardi G.P., Ruoslahti E. (2013). De novo design of a tumor-penetrating peptide. Cancer Res..

[33] Roth L., Agemy L., Kotamraju V.R., Braun G., Teesalu T., Sugahara K.N., Hamzah J., Ruoslahti E. (2012). Transtumoral targeting enabled by a novel neuropilin-binding peptide. Oncogene.

[34] Sugahara K.N., Teesalu T., Karmali P.P., Kotamraju V.R., Agemy L., Girard O.M., Hanahan D., Mattrey R.F., Ruoslahti E. (2009). Tissue-penetrating delivery of compounds and nanoparticles into tumors. Cancer Cell.

[35] He H., Feng M., Hu J., Chen C., Wang J., Wang X., Xu H., Lu J.R. (2012). Designed short RGD peptides for one-pot aqueous synthesis of integrin-binding CdTe and CdZnTe quantum dots. ACS Appl. Mater. Interfaces.

[36] Haubner R., Gratias R., Diefenbach B., Goodman S.L., Jonczyk A., Kessler H. (1996). Structural and functional aspects of RGD-containing cyclic pentapeptides as highly potent and selective integrin αvβ3 antagonists. J. Am. Chem. Soc..

[37] Chen X., Park R., Shahinian A.H., Tohme M., Khankaldyyan V., Bozorgzadeh M.H., Bading J.R., Moats R., Laug W.E., Conti P.S. (2004). 18F-labeled RGD peptide: Initial evaluation for imaging brain tumor angiogenesis. Nucl. Med. Biol..

[38] Van Hagen P.M., Breeman W.A., Bernard H.F., Schaar M., Mooij C.M., Srinivasan A., Schmidt M.A., Krenning E.P., de Jong M. (2000). Evaluation of a radiolabelled cyclic DTPA-RGD analogue for tumour imaging and radionuclide therapy. Int. J. Cancer.

[39] Witt H., Hajdin K., Iljin K., Greiner O., Niggli F.K., Schafer B.W., Bernasconi M. (2009). Identification of a rhabdomyosarcoma targeting peptide by phage display with sequence similarities to the tumour lymphatic-homing peptide LyP-1. Int. J. Cancer.

[40] Kiryushko D., Kofoed T., Skladchikova G., Holm A., Berezin V., Bock E. (2003). A synthetic peptide ligand of neural cell adhesion molecule (NCAM), C3d, promotes neuritogenesis and synaptogenesis and modulates presynaptic function in primary cultures of rat hippocampal neurons. J. Biol. Chem..

[41] Ronn L.C., Olsen M., Ostergaard S., Kiselyov V., Berezin V., Mortensen M.T., Lerche M.H., Jensen P.H., Soroka V., Saffell J.L. (1999). Identification of a neuritogenic ligand of the neural cell adhesion molecule using a combinatorial library of synthetic peptides. Nat. Biotechnol..

[42] Markovsky E., Vax E., Ben-Shushan D., Eldar-Boock A., Shukrun R., Yeini E., Barshack I., Caspi R., Harari-Steinberg O., Pode-Shakked N. (2017). Wilms tumor NCAM-expressing cancer stem cells as potential therapeutic target for polymeric nanomedicine. Mol. Cancer Ther..

[43] Heimann A.S., Gomes I., Dale C.S., Pagano R.L., Gupta A., de Souza L.L., Luchessi A.D., Castro L.M., Giorgi R., Rioli V. (2007). Hemopressin is an inverse agonist of CB1 cannabinoid receptors. Proc. Natl. Acad. Sci. USA.

[44] Li Z., Zhao R., Wu X., Sun Y., Yao M., Li J., Xu Y., Gu J. (2005). Identification and characterization of a novel peptide ligand of epidermal growth factor receptor for targeted delivery of therapeutics. FASEB J..

[45] Lee J.H., Engler J.A., Collawn J.F., Moore B.A. (2001). Receptor mediated uptake of peptides that bind the human transferrin receptor. Eur. J. Biochem..

[46] Lu Y., Jiang W., Wu X., Huang S., Huang Z., Shi Y., Dai Q., Chen J., Ren F., Gao S. (2018). Peptide T7-modified polypeptide with disulfide bonds for targeted delivery of plasmid DNA for gene therapy of prostate cancer. Int. J. Nanomed..

[47] Tang J., Wang Q., Yu Q., Qiu Y., Mei L., Wan D., Wang X., Li M., He Q. (2019). A stabilized retro-inverso peptide ligand of transferrin receptor for enhanced liposome-based hepatocellular carcinoma-targeted drug delivery. Acta Biomater..

[48] Devulapally R., Sekar N.M., Sekar T.V., Foygel K., Massoud T.F., Willmann J.K., Paulmurugan R. (2015). Polymer nanoparticles mediated codelivery of antimiR-10b and antimiR-21 for achieving triple negative breast cancer therapy. ACS Nano.

[49] Stirpe F., Gasperi-Campani A., Barbieri L., Falasca A., Abbondanza A., Stevens W.A. (1983). Ribosome-inactivating proteins from the seeds of *Saponaria officinalis* L. (soapwort), of *Agrostemma githago* L. (corn cockle) and of *Asparagus officinalis* L. (asparagus), and from the latex of *Hura crepitans* L. (sandbox tree). Biochem. J..

[50] Ancheta L.R., Shramm P.A., Bouajram R., Higgins D., Lappi D.A. (2022). Saporin as a commercial reagent: Its uses and unexpected impacts in the biological sciences-tools from the plant kingdom. Toxins.

[51] Huang Y., Shi H., Zhou H., Song X., Yuan S., Luo Y. (2006). The angiogenic function of nucleolin is mediated by vascular endothelial growth factor and nonmuscle myosin. Blood.

[52] Watanabe T., Hirano K., Takahashi A., Yamaguchi K., Beppu M., Fujiki H., Suganuma M. (2010). Nucleolin on the cell surface as a new molecular target for gastric cancer treatment. Biol. Pharm. Bull..

[53] Ding Y., Song N., Liu C., He T., Zhuo W., He X., Chen Y., Song X., Fu Y., Luo Y. (2012). Heat shock cognate 70 regulates the translocation and angiogenic function of nucleolin. Arter. Thromb. Vasc. Biol..

[54] Chen S.C., Hu T.H., Huang C.C., Kung M.L., Chu T.H., Yi L.N., Huang S.T., Chan H.H., Chuang J.H., Liu L.F. (2015). Hepatoma-derived growth factor/nucleolin axis as a novel oncogenic pathway in liver carcinogenesis. Oncotarget.

[55] Krust B., El Khoury D., Nondier I., Soundaramourty C., Hovanessian A.G. (2011). Targeting surface nucleolin with multivalent HB-19 and related Nucant pseudopeptides results in distinct inhibitory mechanisms depending on the malignant tumor cell type. BMC Cancer.

[56] Bates P.J., Laber D.A., Miller D.M., Thomas S.D., Trent J.O. (2009). Discovery and development of the G-rich oligonucleotide AS1411 as a novel treatment for cancer. Exp. Mol. Pathol..

[57] Destouches D., El Khoury D., Hamma-Kourbali Y., Krust B., Albanese P., Katsoris P., Guichard G., Briand J.P., Courty J., Hovanessian A.G. (2008). Suppression of tumor growth and angiogenesis by a specific antagonist of the cell-surface expressed nucleolin. PLoS ONE.

[58] Callebaut C., Blanco J., Benkirane N., Krust B., Jacotot E., Guichard G., Seddiki N., Svab J., Dam E., Muller S. (1998). Identification of V3 loop-binding proteins as potential receptors implicated in the binding of HIV particles to CD4(+) cells. J. Biol. Chem..

[59] Girvan A.C., Teng Y., Casson L.K., Thomas S.D., Juliger S., Ball M.W., Klein J.B., Pierce W.M., Barve S.S., Bates P.J. (2006). AGRO100 inhibits activation of nuclear factor-kappaB (NF-kappaB) by forming a complex with NF-kappaB essential modulator (NEMO) and nucleolin. Mol. Cancer Ther..

[60] Soundararajan S., Chen W., Spicer E.K., Courtenay-Luck N., Fernandes D.J. (2008). The nucleolin targeting aptamer AS1411 destabilizes Bcl-2 messenger RNA in human breast cancer cells. Cancer Res..

[61] Reyes-Reyes E.M., Teng Y., Bates P.J. (2010). A new paradigm for aptamer therapeutic AS1411 action: Uptake by macropinocytosis and its stimulation by a nucleolin-dependent mechanism. Cancer Res..

[62] Xing H., Tang L., Yang X., Hwang K., Wang W., Yin Q., Wong N.Y., Dobrucki L.W., Yasui N., Katzenellenbogen J.A. (2013). Selective Delivery of an Anticancer Drug with Aptamer-Functionalized Liposomes to Breast Cancer Cells in Vitro and in Vivo. J. Mater. Chem. B.

[63] Li S., Jiang Q., Liu S., Zhang Y., Tian Y., Song C., Wang J., Zou Y., Anderson G.J., Han J.Y. (2018). A DNA nanorobot functions as a cancer therapeutic in response to a molecular trigger in vivo. Nat. Biotechnol..

[64] Legrand D., Vigie K., Said E.A., Elass E., Masson M., Slomianny M.C., Carpentier M., Briand J.P., Mazurier J., Hovanessian A.G. (2004). Surface nucleolin participates in both the binding and endocytosis of lactoferrin in target cells. Eur. J. Biochem..

[65] Alibolandi M., Abnous K., Ramezani M., Hosseinkhani H., Hadizadeh F. (2014). Synthesis of AS1411-aptamer-conjugated CdTe quantum dots with high fluorescence strength for probe labeling tumor cells. J. Fluoresc..

[66] Zheng S., Zhang M., Bai H., He M., Dong L., Cai L., Zhao M., Wang Q., Xu K., Li J. (2019). Preparation of AS1411 Aptamer Modified Mn-MoS2 QDs for Targeted MR Imaging and Fluorescence Labelling of Renal Cell Carcinoma. Int. J. Nanomed..

[67] Take M., Tsutsui J., Obama H., Ozawa M., Nakayama T., Maruyama I., Arima T., Muramatsu T. (1994). Identification of nucleolin as a binding protein for midkine (MK) and heparin-binding growth associated molecule (HB-GAM). J. Biochem..

[68] Bulin A.L., Hasan T. (2022). Spatiotemporal Tracking of Different Cell Populations in Cancer Organoid Models for Investigations on Photodynamic Therapy. Methods Mol. Biol..

[69] Vinduska V., Gallops C.E., O’Connor R., Wang Y., Huang X. (2021). Exosomal Surface Protein Detection with Quantum Dots and Immunomagnetic Capture for Cancer Detection. Nanomaterials.

[70] Kuo C.W., Chueh D.Y., Chen P. (2019). Real-time in vivo imaging of subpopulations of circulating tumor cells using antibody conjugated quantum dots. J. Nanobiotech..

[71] Chen Y.Y., Cheng B.R., He Z.B., Wang S.Y., Wang Z.M., Sun M., Song H.B., Fang Y., Chen F.F., Xiong B. (2016). Capture and Identification of Heterogeneous Circulating Tumor Cells Using Transparent Nanomaterials and Quantum Dots-Based Multiplexed Imaging. J. Cancer.

[72] Min H., Jo S.M., Kim H.S. (2015). Efficient capture and simple quantification of circulating tumor cells using quantum dots and magnetic beads. Small.

[73] Lam P.Y., Hillyar C.R., Able S., Vallis K.A. (2016). Synthesis and evaluation of an (18) F-labeled derivative of F3 for targeting surface-expressed nucleolin in cancer and tumor endothelial cells. J. Label. Compd. Radiopharm..

[74] Palmieri D., Richmond T., Piovan C., Sheetz T., Zanesi N., Troise F., James C., Wernicke D., Nyei F., Gordon T.J. (2015). Human anti-nucleolin recombinant immunoagent for cancer therapy. Proc. Natl. Acad. Sci. USA.

[75] Diamantopoulou Z., Gilles M.E., Sader M., Cossutta M., Vallee B., Houppe C., Habert D., Brissault B., Leroy E., Maione F. (2017). Multivalent cationic pseudopeptide polyplexes as a tool for cancer therapy. Oncotarget.

[76] Jia W., Yao Z., Zhao J., Guan Q., Gao L. (2017). New perspectives of physiological and pathological functions of nucleolin (NCL). Life Sci..

[77] Ginisty H., Sicard H., Roger B., Bouvet P. (1999). Structure and functions of nucleolin. J. Cell Sci..

[78] Ugrinova I., Petrova M., Chalabi-Dchar M., Bouvet P. (2018). Multifaceted Nucleolin Protein and Its Molecular Partners in Oncogenesis. Adv. Protein Chem. Struct. Biol..

[79] Koutsioumpa M., Papadimitriou E. (2014). Cell surface nucleolin as a target for anti-cancer therapies. Recent Pat. Anti-Cancer Drug Discov..

[80] Romano S., Fonseca N., Simoes S., Goncalves J., Moreira J.N. (2019). Nucleolin-based targeting strategies for cancer therapy: From targeted drug delivery to cytotoxic ligands. Drug Discov. Today.

[81] Carvalho L.S., Goncalves N., Fonseca N.A., Moreira J.N. (2021). Cancer Stem Cells and Nucleolin as Drivers of Carcinogenesis. Pharmaceuticals.

[82] Ferrara B., Belbekhouche S., Habert D., Houppe C., Vallee B., Bourgoin-Voillard S., Cohen J.L., Cascone I., Courty J. (2021). Cell surface nucleolin as active bait for nanomedicine in cancer therapy: A promising option. Nanotechnology.

[83] Tonello F., Massimino M.L., Peggion C. (2022). Nucleolin: A cell portal for viruses, bacteria, and toxins. Cell Mol. Life Sci..

[84] Karamchand L., Kim G., Wang S., Hah H.J., Ray A., Jiddou R., Koo Lee Y.E., Philbert M.A., Kopelman R. (2013). Modulation of hydrogel nanoparticle intracellular trafficking by multivalent surface engineering with tumor targeting peptide. Nanoscale.

[85] Su P.Y., Wang Y.F., Huang S.W., Lo Y.C., Wang Y.H., Wu S.R., Shieh D.B., Chen S.H., Wang J.R., Lai M.D. (2015). Cell surface nucleolin facilitates enterovirus 71 binding and infection. J. Virol..

[86] Hovanessian A.G., Puvion-Dutilleul F., Nisole S., Svab J., Perret E., Deng J.S., Krust B. (2000). The cell-surface-expressed nucleolin is associated with the actin cytoskeleton. Exp. Cell Res..

[87] Nohira N., Shinji S., Nakamura S., Nihashi Y., Shimosato T., Takaya T. (2021). Myogenetic oligodeoxynucleotides as anti-nucleolin aptamers inhibit the growth of embryonal rhabdomyosarcoma cells. bioRxiv.

[88] Willmer T., Damerell V., Smyly S., Sims D., Du Toit M., Ncube S., Sinkala M., Govender D., Sturrock E., Blackburn J.M. (2021). Targeting the oncogenic TBX3:nucleolin complex to treat multiple sarcoma subtypes. Am. J. Cancer Res..

[89] Sims D., Maranyane H.M., Damerell V., Govender D., Isaacs A.W., Peres J., Prince S. (2020). The c-Myc/AKT1/TBX3 Axis Is Important to Target in the Treatment of Embryonal Rhabdomyosarcoma. Cancers.

[90] Rammal G., Fahs A., Kobeissy F., Mechref Y., Zhao J., Zhu R., Diab-Assaf M., Saab R., Ghayad S.E. (2019). Proteomic Profiling of Rhabdomyosarcoma-Derived Exosomes Yield Insights into Their Functional Role in Paracrine Signaling. J. Proteome Res..

[91] Benedetti E., Antonosante A., d’Angelo M., Cristiano L., Galzio R., Destouches D., Florio T.M., Dhez A.C., Astarita C., Cinque B. (2015). Nucleolin antagonist triggers autophagic cell death in human glioblastoma primary cells and decreased in vivo tumor growth in orthotopic brain tumor model. Oncotarget.

[92] Gilles M.E., Maione F., Cossutta M., Carpentier G., Caruana L., Di Maria S., Houppe C., Destouches D., Shchors K., Prochasson C. (2016). Nucleolin Targeting Impairs the Progression of Pancreatic Cancer and Promotes the Normalization of Tumor Vasculature. Cancer Res..

[93] Fonseca N.A., Gregório A.C., Mendes V.M., Lopes R., Abreu T., Gonçalves N., Manadas B., Lacerda M., Figueiredo P., Pereira M. (2021). GMP-grade nanoparticle targeted to nucleolin downregulates tumor molecular signature, blocking growth and invasion, at low systemic exposure. Nano Today.

[94] Brignole C., Bensa V., Fonseca N.A., Del Zotto G., Bruno S., Cruz A.F., Malaguti F., Carlini B., Morandi F., Calarco E. (2021). Cell surface Nucleolin represents a novel cellular target for neuroblastoma therapy. J. Exp. Clin. Cancer Res..

[95] Jaaks P., D’Alessandro V., Grob N., Buel S., Hajdin K., Schafer B.W., Bernasconi M. (2016). The Proprotein Convertase Furin Contributes to Rhabdomyosarcoma Malignancy by Promoting Vascularization, Migration and Invasion. PLoS ONE.

[96] Jaaks P., Meier G., Alijaj N., Brack E., Bode P., Koscielniak E., Wachtel M., Schafer B.W., Bernasconi M. (2016). The proprotein convertase furin is required to maintain viability of alveolar rhabdomyosarcoma cells. Oncotarget.

[97] Phimister E.G., Culverwell A., Patel K., Kemshead J.T. (1994). Tissue-specific expression of neural cell adhesion molecule (NCAM) may allow differential diagnosis of neuroblastoma from embryonal rhabdomyosarcoma. Eur. J. Cancer.

[98] Oesch S., Walter D., Wachtel M., Pretre K., Salazar M., Guzman M., Velasco G., Schafer B.W. (2009). Cannabinoid receptor 1 is a potential drug target for treatment of translocation-positive rhabdomyosarcoma. Mol. Cancer Ther..

[99] De Giovanni C., Landuzzi L., Palladini A., Nicoletti G., Nanni P., Lollini P.L. (2021). HER Tyrosine Kinase Family and Rhabdomyosarcoma: Role in Onset and Targeted Therapy. Cells.

[100] Ganti R., Skapek S.X., Zhang J., Fuller C.E., Wu J., Billups C.A., Breitfeld P.P., Dalton J.D., Meyer W.H., Khoury J.D. (2006). Expression and genomic status of EGFR and ErbB-2 in alveolar and embryonal rhabdomyosarcoma. Mod. Pathol..

[101] Falvo E., Damiani V., Conti G., Boschi F., Messana K., Giacomini P., Milella M., De Laurenzi V., Morea V., Sala G. (2021). High activity and low toxicity of a novel CD71-targeting nanotherapeutic named The-0504 on preclinical models of several human aggressive tumors. J. Exp. Clin. Cancer Res..

[102] Oh F., Todhunter D., Taras E., Vallera D.A., Borgatti A. (2018). Targeting EGFR and uPAR on human rhabdomyosarcoma, osteosarcoma, and ovarian adenocarcinoma with a bispecific ligand-directed toxin. Clin. Pharmacol..

[103] Pilbeam K., Wang H., Taras E., Bergerson R.J., Ettestad B., DeFor T., Borgatti A., Vallera D.A., Verneris M.R. (2018). Targeting pediatric sarcoma with a bispecific ligand immunotoxin targeting urokinase and epidermal growth factor receptors. Oncotarget.

[104] Kessler T., Baumeier A., Brand C., Grau M., Angenendt L., Harrach S., Stalmann U., Schmidt L.H., Gosheger G., Hardes J. (2018). Aminopeptidase N (CD13): Expression, Prognostic Impact, and Use as Therapeutic Target for Tissue Factor Induced Tumor Vascular Infarction in Soft Tissue Sarcoma. Transl. Oncol..

[105] Brohl A.S., Sindiri S., Wei J.S., Milewski D., Chou H.C., Song Y.K., Wen X., Kumar J., Reardon H.V., Mudunuri U.S. (2021). Immuno-transcriptomic profiling of extracranial pediatric solid malignancies. Cell Rep..

[106] Hayes M.N., McCarthy K., Jin A., Oliveira M.L., Iyer S., Garcia S.P., Sindiri S., Gryder B., Motala Z., Nielsen G.P. (2018). Vangl2/RhoA Signaling Pathway Regulates Stem Cell Self-Renewal Programs and Growth in Rhabdomyosarcoma. Cell Stem. Cell.

