# Supplementary material for: Quantum Dot-Based Screening Identifies F3 Peptide and Reveals Cell Surface Nucleolin as a Therapeutic Target for Rhabdomyosarcoma
Source: Cancers (Basel). 2022 Oct 14;14(20):5048. doi: 10.3390/cancers14205048 (PMC9600270; doi:10.3390/cancers14205048)
Supplement: Supplementary file 1 [file cancers-14-05048-s001.zip › cancers-1936022-supplementary.pdf]

Supplementary Materials:

# Table S1. Peptides used in this study

| Name | Name in original reference | N- terminal modification | Sequence | C-terminal modification | Mw | Purity | Source |
|---|---|---|---|---|---|---|---|
| **Biotin-TAT (47-57)** | TAT | biotin- | YGRKKRRQRRR | OH | 1786.2 | | Eurogentec/Anaspec |
| | | | | | | | |
| **CmR** | CmR | biotin-{Ahx}{Ahx}{Ahx} | KRDRGGGCMGTINTATAAC-NH2 | NH2 | 2445.93 | 97% | GenScript |
| **cRADyK** | cyclic RAD | | Cyclo[RAD-D-Phe-K-biotin-PEG-PEG] | | 1134.33 | 95% | Peptides International |
| **cRGDyK** | cyclic RGD | | Cyclo[RGD-D-Phe-K-biotin-PEG-PEG] | | 1120.3 | 97% | Peptides International |
| **F3** | F3 | biotin-{Ahx}{Ahx}{Ahx} | EPQRRSARLSAKPAPPKPEPKPKKAPAKK | NH2 | 3754.61 | 97% | GenScript |
| **GE11** | GE11 | biotin-{Ahx}{Ahx}{Ahx} | YHWYGYTPQNVI | NH2 | 2105.46 | 97% | GenScript |
| **Hemopressin** | Hemopressin (HP) | biotin-{Ahx}{Ahx}{Ahx} | PVNFKFLSH | NH2 | 1653.04 | 96% | GenScript |
| **iNGR** | iNGR | biotin-{Ahx}{Ahx}{Ahx} | CRNGRGPDC | NH2 | 1539.9 | 98% | GenScript |
| **iRGD** | iRGD | biotin-{Ahx}{Ahx}{Ahx} | CRGDKGPDC | NH2 | 1512.87 | 97% | GenScript / solubility |
| **iso-d-T7** | T7 (D-isomer) | biotin-{Ahx}{Ahx}{Ahx} | {d-HIS}{d-ARG}{d-PRO}{d-TYR}{d-ILE}{d-ALA}{d-HIS} | NH2 | 1457.79 | 98% | GenScript |
| **Lyp1** | Lyp-1 | biotin-{Ahx}{Ahx}{Ahx} | CGNKRTRGC | NH2 | 1556.95 | 96% | GenScript / solubility |
| **NGR18** | NGR-2C-TNF1–11 | NH2 | CNGRCGVRSSSRTPSDKY | {Ahx}{Ahx}{Ahx}-K-biotin | 2707.14 | 96% | GenScript |
| **NGR5** | NGR-5 | NH2 | YNGRT | {Ahx}{Ahx}{Ahx}-K-biotin | 1345.66 | 97% | GenScript / solubility |
| **NTP** | C3, NTP (NCAM-1 targeting peptide) | biotin-{Ahx}{Ahx}{Ahx} | ASKKPKRNIKA | NH2 | 1805.27 | 98% | GenScript |
| **pAGP** | pAGP | biotin-{Ahx}{Ahx}{Ahx} | CAGPRTRRC | NH2 | 1582 | 95% | GenScript |
| **RMS-I** | RMS-I | biotin-{Ahx}{Ahx}{Ahx} | CQQSNRGDRKRC | NH2 | 2013.43 | 99% | GenScript |
| **RMS-II** | RMS-II | biotin-{Ahx}{Ahx}{Ahx} | CMGNKRSAKRPC | NH2 | 1913.43 | 96% | GenScript |
| **RMS-P3** | RMS-P3 | biotin-{Ahx}{Ahx}{Ahx} | CMGTINTRTRRC | NH2 | 1974.48 | 95% | GenScript |
| **RMS-P3-3G** | | biotin-{Ahx}{Ahx}{Ahx} | GGGCMGTINTRTRRC | NH2 | 2145.59 | 96% | GenScript |
| **shTmR (aka TmRggg)** | | biotin-{Ahx}{Ahx}{Ahx} | KRDRCMGTINTRTRRC | NH2 | 2530.1 | 95% | GenScript |
| **T7** | T7 | biotin-{Ahx}{Ahx}{Ahx} | HAIYPRH | NH2 | 1457.77 | 98% | GenScript |
| **tLyp1** | tLyp-1 | biotin-{Ahx}{Ahx}{Ahx} | CGNKRTR | NH2 | 1398.8 | 95% | GenScript |
| **TmR** | TmR | biotin-{Ahx}{Ahx}{Ahx} | KRDRGGGCMGTINTRTRRC | NH2 | 2701.26 | 95% | GenScript |
| **uPA** | U11 | biotin-{Ahx}{Ahx}{Ahx} | VSNKYFSNIHWGC | NH2 | 2119.5 | 96% | GenScript |

**Table S2. Published work supporting expression of selected targets in RMS cell lines or tumors.**

| Target | Uniprot | Gene | Evidence in the literature |
|---|---|---|---|
| Furin | P09958 | FURIN | RMS patient samples and cell lines [1-3] |
| Neural Cell Adhesion Molecule 1 / CD56 (NCAM-1) | P13591 | NCAM1 | Preferentially expressed in RMS [4].<br>This publication |
| Cannabinoid Receptor 1 / CB1 | P21554 | CNR1 | Gene expression regulated by PAX3/FOXO1 [5] |
| Epidermal Growth Factor Receptor c-ErbB-1 | P00533 | EGFR | Reviewed in [6]. E.g., expression in 31/66 (47%) cases; FN: 26/34, 76%, FP: 5/32, 16% [7] |
| Transferrin Receptor 1 / CD71 (TFR1) | P02786 | TFRC | Overexpression in A204 cells [8] |
| Urokinase Plasminogen Activator Receptor (uPAR) | Q03405 | PLAUR | uPAR targeted in RD RMS cells by bispecific uPAR/EGFR immunotoxin [9] and in Rh30 RMS cells [10]. |
| Nucleolin (Protein C23) | P19338 | NCL | RD cell line [11,12]<br>RMS PDX (n=5) [11]<br>FN-RMS / ERMS cells [13]<br>This publication |
| Aminopeptidase N / CD13 (APN) | P15144 | ANPEP | Expression in soft tissue sarcomas [14] |
| Complement component 1 Q subcomponent-binding protein, mitochondrial, p32 | Q07021 | C1QBP | No direct evidence in the literature |
| Neuropilin-1 | Q9H2D9 | NRP1 | No direct evidence in the literature |
| Integrin $\alpha_v\beta_3$ | P06756<br>P05106 | ITAV<br>ITGB3 | Expression in Rh30 cells [15,16] and RD cells [17] |

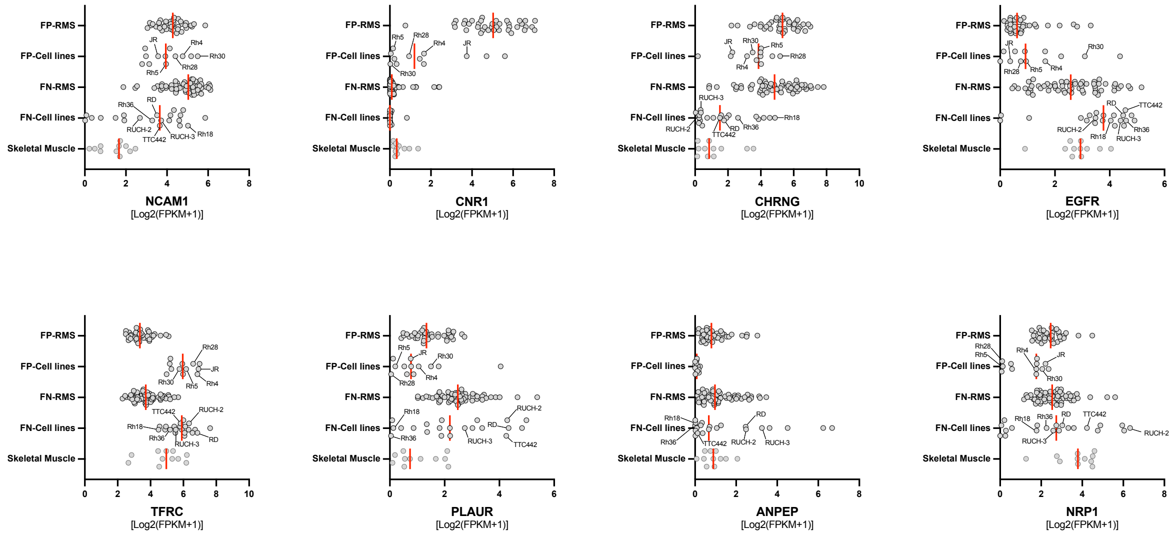


**Figure S1. Targeted Receptors Expression in RMS cell lines, tumors, and skeletal muscles.** Whole transcriptome sequencing data for cell surface proteins were extracted from Brohl et al. [18]. The data set from the online Supplementary Material Excel file mmc4.xlsx - S3A.2.Ex_CellSurfaceProteins contains data from 60 FN RMS tumors, 38 FP-RMS tumors, 21 FN-RMS cell lines, 12 FP-RMS cell lines, 11 skeletal muscles tissues Log2 values of Fragments Per Kilobase Million (FPKM) are indicated for each selected gene. Highlighted are RMS cell lines available in our laboratory. When compared to skeletal muscles, NCAM1, CNR1, CHRNG, PLAUR, present higher transcripts numbers in RMS samples. Transcripts expression of EGFR, TFRC, NRP1, ANPEP is not strikingly higher in RMS samples.

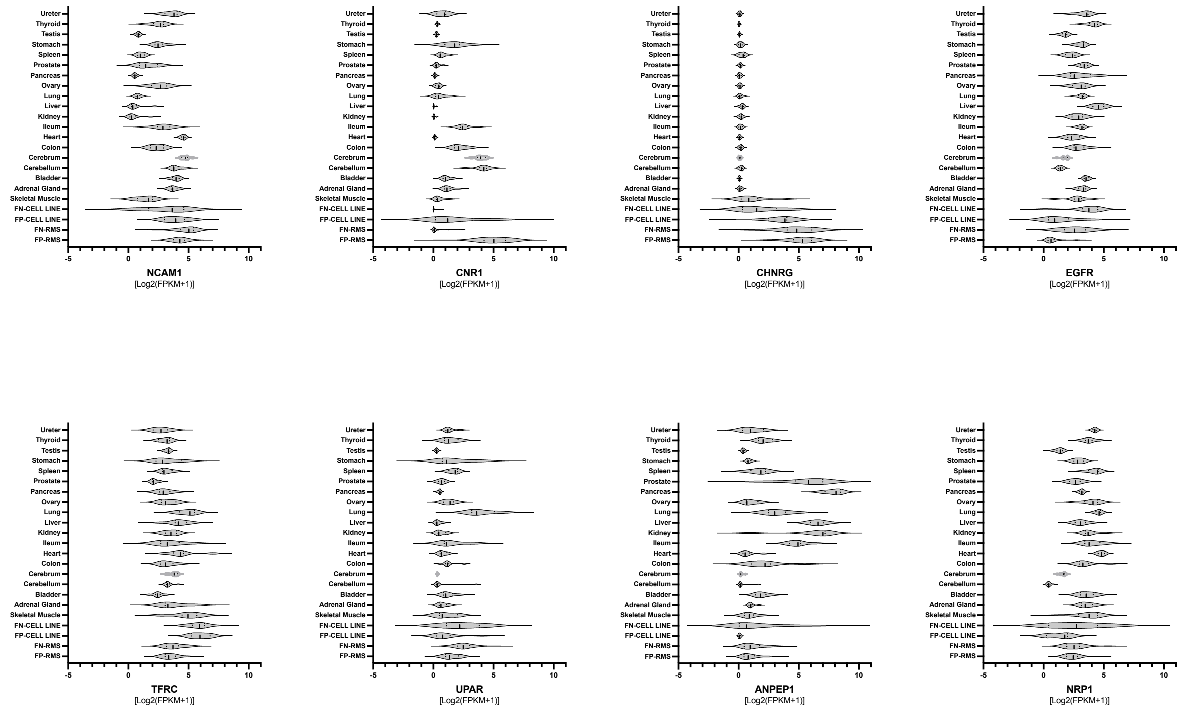


**Figure S2. Targeted Receptors Expression in RMS cell lines, tumors, and normal tissues.** Whole transcriptome sequencing data for cell surface proteins were extracted from Brohl et al. [18]. The data set from the online Supplementary Material Excel file mmc4.xlsx - S3A.2.Ex_CellSurfaceProteins contains data from 60 FN RMS tumors, 38 FP-RMS tumors, 21 FN-RMS cell lines, 12 FP-RMS cell lines, 11 skeletal muscles tissues), and additional control tissues. Log2 values of Fragments Per Kilobase Million (FPKM) are indicated for each selected gene. Transcripts in 19 control tissues are shown. Striking is the almost complete absence of expression of CHNRG in all tissues analyzed, except for skeletal muscles, where expression is however lower than in RMS tumors and cell lines.

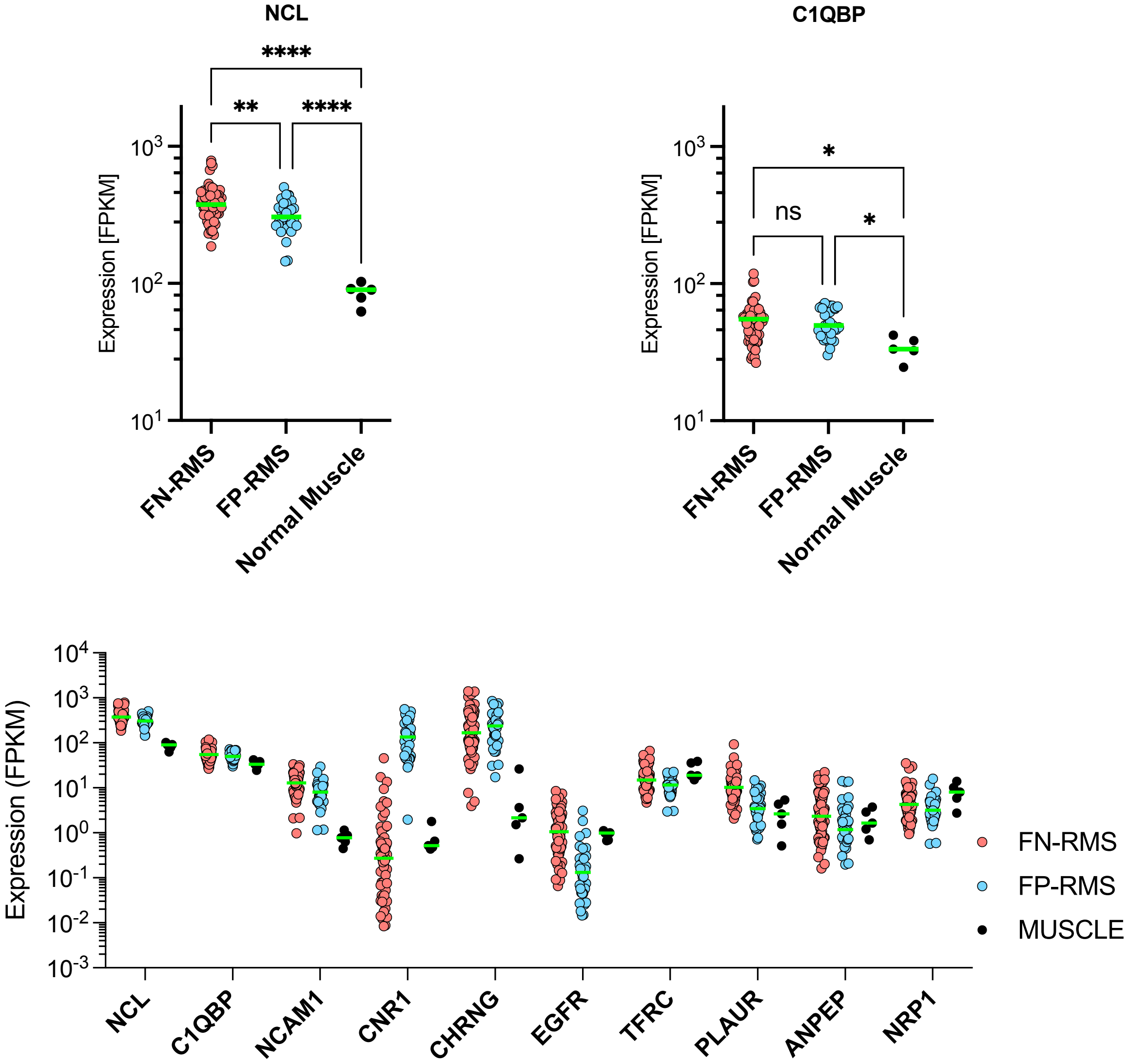


**Figure S3. Expression of selected surface targets including nucleolin and p32 in RMS cell lines, tumors, and skeletal muscle.** Since Nucleolin and p32 are not included in Fig.S1 and Fig.S2 as they are not 'classical' surface proteins, we have extracted expression data from a published RNA sequencing of 34 FP-RMS, 66 FN-RMS, and 5 normal skeletal muscles where transcripts were quantified by RNA sequencing [19] (GEO accession number GSE108022). Displayed are Fragments Per Kilobase Million (FPKM).

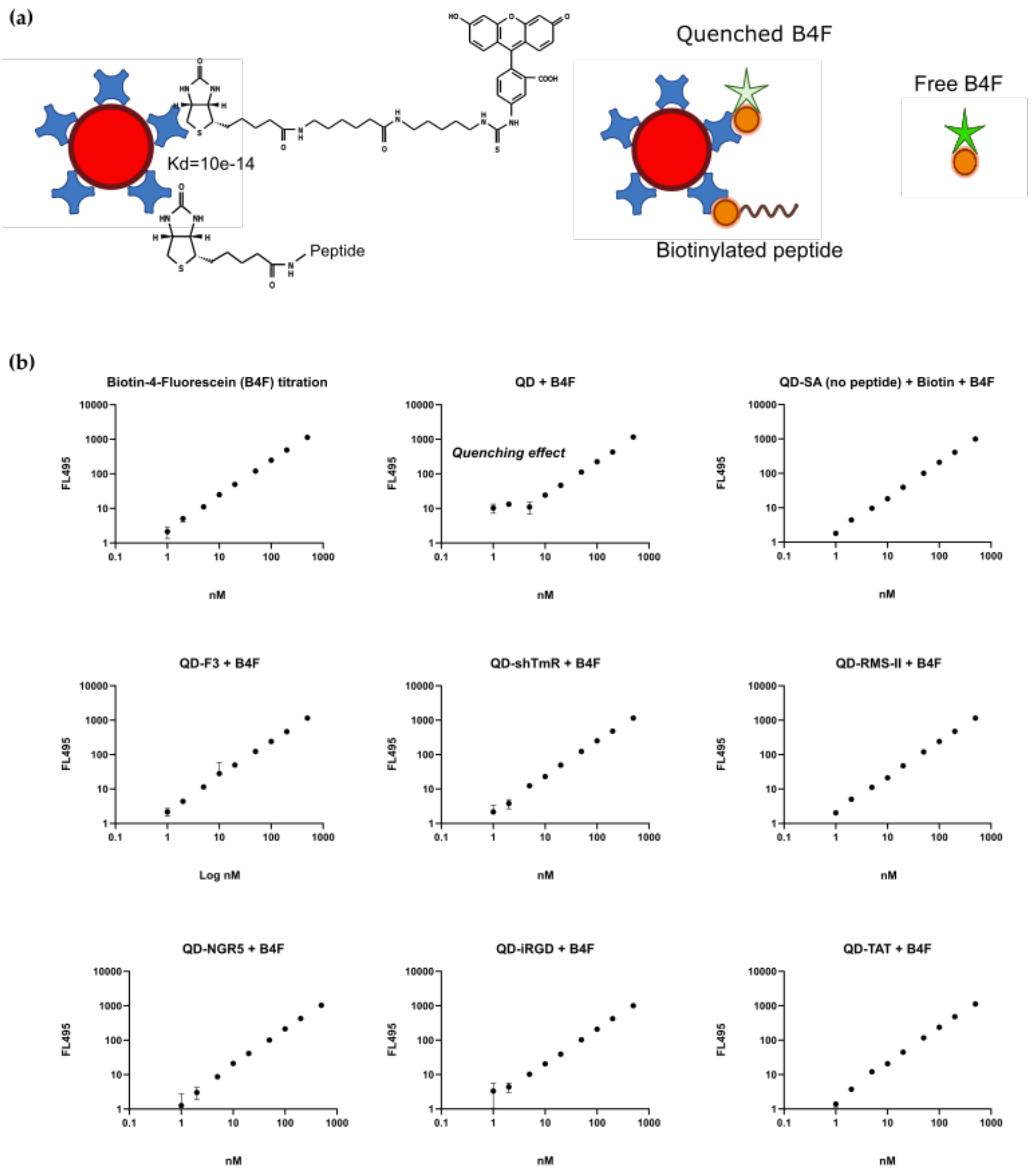


**Figure S4. Quality control for conjugation of streptavidin-QD and biotin-Peptides by B4F quenching**. **(a)** Schematic illustration of the quenching process taking place when Biotin-4-FITC binds to QDs. **(b)** Representative results obtained for the different biotinylated peptides. When Biotin-4-Fluorescein was incubated from 1 to 500nM with QD-SA alone (QD + B4F) the quenching was visible at concentrations lower than 10nM. Preincubated of QD-SA with free biotin abrogated this effect, as well as the conjugation with biotinylated peptides, indicating successful conjugation.

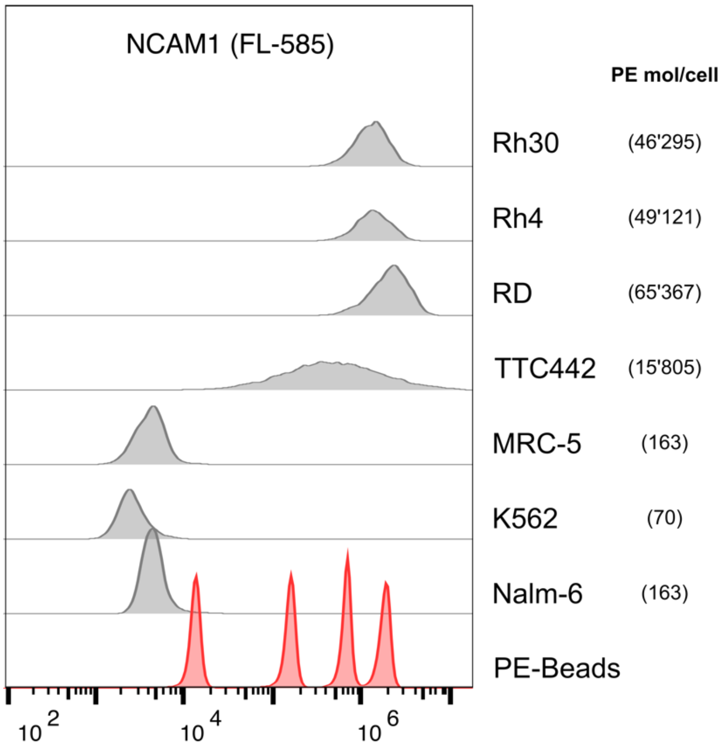


**Figure S5. Surface expression of NCAM1 on RMS cell lines and control cells.** Cells were detached by Accutase and after washing were incubated with PE-conjugated anti-NCAM1. Quantibrite PE-Beads (340495, BS Biosciences) were used according to the manufacturer instructions. The four peaks correspond to (from left to right) 1700, 14200, 39400, 133400 molecules PE per bead. Quantification was performed by assuming one molecule PE per antibody.

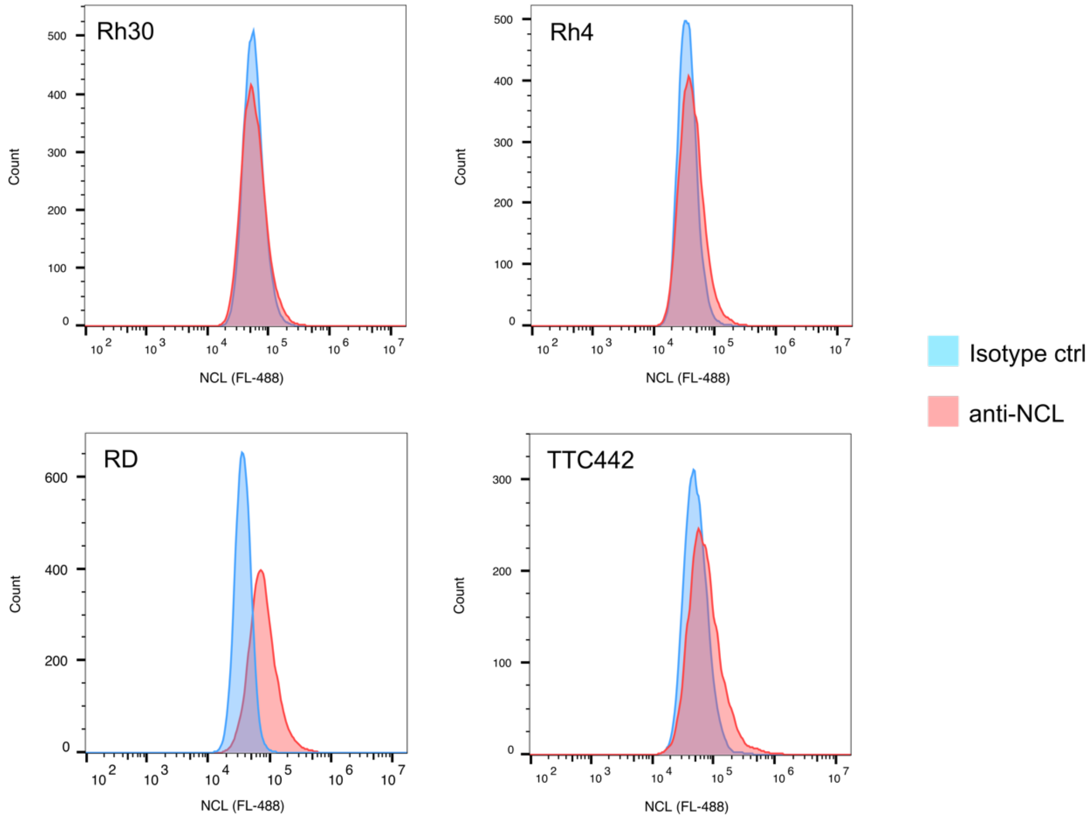


**Figure S6. Surface detection of Nucleolin on RMS cells.** Cells were detached with Accutase and after washing in PBS/2%BSA 100'000 were incubated with anti-Nucleolin polyclonal Rabbit Antibody (N2662, Sigma) diluted 1/50, for 1h at 4°C. After washing, secondary antibody Goat anti-Rabbit IgG (H+L) Cross-adsorbed, Alexa488 (A11008, Thermofisher) 1/500 was incubated for 30 min at 4°C. Rabbit IgG Isotype Control (02-6102, Thermofisher) was used as control. After washing, cells were fixed with 1%PFA/PBS and measured by flow cytometry. No consistent shift could be observed for all cell lines analyzed. Only RD cells did show a clear increased fluorescence upon incubation with anti-Nucleolin antibody, as compared to isotype control.

## References


1. Jaaks, P.; D'Alessandro, V.; Grob, N.; Buel, S.; Hajdin, K.; Schafer, B.W.; Bernasconi, M. The Proprotein Convertase Furin Contributes to Rhabdomyosarcoma Malignancy by Promoting Vascularization, Migration and Invasion. *PLoS One* **2016**, *11*, e0161396, doi:10.1371/journal.pone.0161396.
2. Jaaks, P.; Meier, G.; Alijaj, N.; Brack, E.; Bode, P.; Koscielniak, E.; Wachtel, M.; Schafer, B.W.; Bernasconi, M. The proprotein convertase furin is required to

maintain viability of alveolar rhabdomyosarcoma cells. *Oncotarget* **2016**, *7*, 76743-76755, doi:10.18632/oncotarget.11648.
3. Hajdin, K.; D'Alessandro, V.; Niggli, F.K.; Schafer, B.W.; Bernasconi, M. Furin targeted drug delivery for treatment of rhabdomyosarcoma in a mouse model. *PLoS One* **2010**, *5*, e10445, doi:10.1371/journal.pone.0010445.
4. Phimister, E.G.; Culverwell, A.; Patel, K.; Kemshead, J.T. Tissue-specific expression of neural cell adhesion molecule (NCAM) may allow differential diagnosis of neuroblastoma from embryonal rhabdomyosarcoma. *Eur J Cancer* **1994**, *30A*, 1552-1558, doi:10.1016/0959-8049(94)00289-h.
5. Oesch, S.; Walter, D.; Wachtel, M.; Pretre, K.; Salazar, M.; Guzman, M.; Velasco, G.; Schafer, B.W. Cannabinoid receptor 1 is a potential drug target for treatment of translocation-positive rhabdomyosarcoma. *Mol Cancer Ther* **2009**, *8*, 1838-1845, doi:10.1158/1535-7163.MCT-08-1147.
6. De Giovanni, C.; Landuzzi, L.; Palladini, A.; Nicoletti, G.; Nanni, P.; Lollini, P.L. HER Tyrosine Kinase Family and Rhabdomyosarcoma: Role in Onset and Targeted Therapy. *Cells* **2021**, *10*, doi:10.3390/cells10071808.
7. Ganti, R.; Skapek, S.X.; Zhang, J.; Fuller, C.E.; Wu, J.; Billups, C.A.; Breitfeld, P.P.; Dalton, J.D.; Meyer, W.H.; Khoury, J.D. Expression and genomic status of EGFR and ErbB-2 in alveolar and embryonal rhabdomyosarcoma. *Mod Pathol* **2006**, *19*, 1213-1220, doi:10.1038/modpathol.3800636.
8. Falvo, E.; Damiani, V.; Conti, G.; Boschi, F.; Messana, K.; Giacomini, P.; Milella, M.; De Laurenzi, V.; Morea, V.; Sala, G.; et al. High activity and low toxicity of a novel CD71-targeting nanotherapeutic named The-0504 on preclinical models of several human aggressive tumors. *J Exp Clin Cancer Res* **2021**, *40*, 63, doi:10.1186/s13046-021-01851-8.
9. Oh, F.; Todhunter, D.; Taras, E.; Vallera, D.A.; Borgatti, A. Targeting EGFR and uPAR on human rhabdomyosarcoma, osteosarcoma, and ovarian adenocarcinoma with a bispecific ligand-directed toxin. *Clin Pharmacol* **2018**, *10*, 113-121, doi:10.2147/CPAA.S160262.
10. Pilbeam, K.; Wang, H.; Taras, E.; Bergerson, R.J.; Ettestad, B.; DeFor, T.; Borgatti, A.; Vallera, D.A.; Verneris, M.R. Targeting pediatric sarcoma with a bispecific ligand immunotoxin targeting urokinase and epidermal growth factor receptors. *Oncotarget* **2018**, *9*, 11938-11947, doi:10.18632/oncotarget.21187.
11. Willmer, T.; Damerell, V.; Smyly, S.; Sims, D.; Du Toit, M.; Ncube, S.; Sinkala, M.; Govender, D.; Sturrock, E.; Blackburn, J.M.; et al. Targeting the oncogenic TBX3:nucleolin complex to treat multiple sarcoma subtypes. *Am J Cancer Res* **2021**, *11*, 5680-5700.
12. Su, P.Y.; Wang, Y.F.; Huang, S.W.; Lo, Y.C.; Wang, Y.H.; Wu, S.R.; Shieh, D.B.; Chen, S.H.; Wang, J.R.; Lai, M.D.; et al. Cell surface nucleolin facilitates enterovirus 71 binding and infection. *J Virol* **2015**, *89*, 4527-4538, doi:10.1128/JVI.03498-14.

13. Nohira, N.; Shinji, S.; Nakamura, S.; Nihashi, Y.; Shimosato, T.; Takaya, T. Myogenetic oligodeoxynucleotides as anti-nucleolin aptamers inhibit the growth of embryonal rhabdomyosarcoma cells. *bioRxiv* **2021**, 2021.2010.2018.464889, doi:10.1101/2021.10.18.464889.
14. Kessler, T.; Baumeier, A.; Brand, C.; Grau, M.; Angenendt, L.; Harrach, S.; Stalmann, U.; Schmidt, L.H.; Gosheger, G.; Hardes, J.; et al. Aminopeptidase N (CD13): Expression, Prognostic Impact, and Use as Therapeutic Target for Tissue Factor Induced Tumor Vascular Infarction in Soft Tissue Sarcoma. *Transl Oncol* **2018**, *11*, 1271-1282, doi:10.1016/j.tranon.2018.08.004.
15. Rengaswamy, V.; Zimmer, D.; Suss, R.; Rossler, J. RGD liposome-protamine-siRNA (LPR) nanoparticles targeting PAX3-FOXO1 for alveolar rhabdomyosarcoma therapy. *J Control Release* **2016**, *235*, 319-327, doi:10.1016/j.jconrel.2016.05.063.
16. Scherzinger-Laude, K.; Schonherr, C.; Lewrick, F.; Suss, R.; Francese, G.; Rossler, J. Treatment of neuroblastoma and rhabdomyosarcoma using RGD-modified liposomal formulations of patupilone (EPO906). *International Journal of Nanomedicine* **2013**, *8*, 2197-2211, doi:10.2147/IJN.S44025.
17. Witt, H.; Hajdin, K.; Iljin, K.; Greiner, O.; Niggli, F.K.; Schafer, B.W.; Bernasconi, M. Identification of a rhabdomyosarcoma targeting peptide by phage display with sequence similarities to the tumour lymphatic-homing peptide LyP-1. *Int J Cancer* **2009**, *124*, 2026-2032, doi:10.1002/ijc.24170.
18. Brohl, A.S.; Sindiri, S.; Wei, J.S.; Milewski, D.; Chou, H.C.; Song, Y.K.; Wen, X.; Kumar, J.; Reardon, H.V.; Mudunuri, U.S.; et al. Immuno-transcriptomic profiling of extracranial pediatric solid malignancies. *Cell Rep* **2021**, *37*, 110047, doi:10.1016/j.celrep.2021.110047.
19. Hayes, M.N.; McCarthy, K.; Jin, A.; Oliveira, M.L.; Iyer, S.; Garcia, S.P.; Sindiri, S.; Gryder, B.; Motala, Z.; Nielsen, G.P.; et al. Vangl2/RhoA Signaling Pathway Regulates Stem Cell Self-Renewal Programs and Growth in Rhabdomyosarcoma. *Cell Stem Cell* **2018**, *22*, 414-427 e416, doi:10.1016/j.stem.2018.02.002.
